# Human milk extracellular vesicles enhance muscle growth and physical performance of immature mice associating with Akt/mTOR/p70s6k signaling pathway

**DOI:** 10.1186/s12951-023-02043-6

**Published:** 2023-08-29

**Authors:** Zitong Meng, Dong Zhou, Dan Lv, Quan Gan, Yuxiao Liao, Zhao Peng, Xiaolei Zhou, Shiyin Xu, Penglong Chi, Zhipeng Wang, Andreas K. Nüssler, Xuefeng Yang, Liegang Liu, Dongrui Deng, Wei Yang

**Affiliations:** 1 Department of Nutrition and Food Hygiene, Hubei Key Laboratory of Food Nutrition and Safety, Tongji Medical College, Huazhong University of Science and Technology, Hangkong Road 13, Wuhan, 430030 China; 2 Department of Nutrition and Food Hygiene and MOE Key Lab of Environment and Health, School of Public Health, Tongji Medical College, Huazhong University of Science and Technology, Hangkong Road 13, Wuhan, 430030 China; 3 Department of Obstetrics, Maternal and Child Health Hospital of Hubei Province, 745 Wuluo Road, Wuhan, 430000 China; 4 Department of Critical Care Medicine, Maternal and Child Health Hospital of Hubei Province, 745 Wuluo Road, Wuhan, 430000 China; 5 Department of Obstetrics and Gynecology, Tongji Hospital, Tongji Medical College, Huazhong University of Science and Technology, Jiefang Avenue 1095, Wuhan, 430030 Hubei China; 6 Department of Traumatology, BG Trauma Center, University of Tübingen, Schnarrenbergstr. 95, 72076 Tübingen, Germany

**Keywords:** HME, BME, Amino acid composition, Muscle performance, Muscle growth

## Abstract

Extracellular vesicles (EVs) play an important role in human and bovine milk composition. According to excellent published studies, it also exerts various functions in the gut, bone, or immune system. However, the effects of milk-derived EVs on skeletal muscle growth and performance have yet to be fully explored. Firstly, the current study examined the amino acids profile in human milk EVs (HME) and bovine milk EVs (BME) using targeted metabolomics. Secondly, HME and BME were injected in the quadriceps of mice for four weeks (1 time/3 days). Then, related muscle performance, muscle growth markers/pathways, and amino acids profile were detected or measured by grip strength analysis, rotarod performance testing, Jenner-Giemsa/H&E staining, Western blotting, and targeted metabolomics, respectively. Finally, HME and BME were co-cultured with C2C12 cells to detect the above-related indexes and further testify relative phenomena. Our findings mainly demonstrated that HME and BME significantly increase the diameter of C2C12 myotubes. HME treatment demonstrates higher exercise performance and muscle fiber densities than BME treatment. Besides, after KEGG and correlation analyses with biological function after HME and BME treatment, results showed L-Ornithine acts as a “notable marker” after HME treatment to affect mouse skeletal muscle growth or functions. Otherwise, L-Ornithine also significantly positively correlates with the activation of the AKT/mTOR pathway and myogenic regulatory factors (MRFs) and can also be observed in muscle and C2C12 cells after HME treatment. Overall, our study not only provides a novel result for the amino acid composition of HME and BME, but the current study also indicates the advantage of human milk on skeletal muscle growth and performance.

**Supplementary Information:**

The online version contains supplementary material available at 10.1186/s12951-023-02043-6.

## Highlights

Different amino acid distributions between HME and BME.

HME has higher Creatine-phosphate and L-Ornithine.

HME injection improves muscle growth/performance associated AKT-mTOR-p70s6k signaling.

L-Ornithine in HME may be a crucial factor for skeletal muscle function.

**Supplementary Information:**

The online version contains supplementary material available at 10.1186/s12951-023-02043-6.

## Introduction

Skeletal muscle is the largest tissue in the body and plays a pivotal role in movement, posture, and energy metabolism [1, 2]. During childhood, skeletal muscle undergoes rapid growth and development, which is critical for overall growth and development, as it provides the necessary strength for movement and supports bone development [3]. Skeletal muscle also plays a crucial role in maintaining energy balance by regulating glucose metabolism and insulin sensitivity [4, 5]. Moreover, enhancing or maintaining skeletal muscle function or growth can prevent various diseases in children. For instance, muscle strength has been closely linked to metabolic disorders and diseases, such as obesity and insulin resistance in children [6, 7]. Nutritional intervention for skeletal muscle function also may profoundly affect physical activity and quality of life [8].

Milk, a nutritionally complex fluid secreted by the mammary glands of female mammals, contains valuable lipids, proteins and amino acids that play essential roles in skeletal muscle protein synthesis. Milk also contains different bioactive compounds, some offering potential health benefits, notably extracellular vesicles (EVs). These membrane-bound small vesicles carry various informative cargoes (substances) and have potential roles in cell communication, metabolism, and regulating nutrition absorption that has triggered the interest of researchers in recent years [9, 10]. An excellent previous study also demonstrated that bovine milk whey-derived EVs positively function in the protein synthesis of C2C12 cells [11]. However, animal studies have yielded contrary results [12, 13]. These different results possibly link with the differential distribution of milk extracellular vesicles in various organs or tissues [14, 15]. Therefore, it is necessary to investigate further milk extracellular vesicles’ direct effects on skeletal muscle in animal models to clarify their concrete roles or/and mechanism in muscle growth or other functions.

In recent years, researchers have illustrated and identified EVs in milk from various mammalian species. These EVs have been found to contain biologically active molecules that mediate a wide range of physiological functions [16, 17]. The composition of these molecules differs between milk-derived EVs from different species, perhaps due to differences in the cells’ external environment or cellular state [18]. For example, human milk-derived EVs (HME) are highly enriched in immune-related proteins and miRNA that support the development and maturation of the infant immune system [19]. On the other hand, bovine milk-derived EVs (BME) have been shown to possess anti-inflammatory and antioxidant properties that may provide potential benefits for human health [20]. Although research on milk EVs currently focuses on mRNA, miRNA, and lipids, the role of amino acids in EVs remains unclear [16, 20, 21]. Amino acids are essential to life and exert as substrates for protein synthesis. For instance, branched-chain amino acids (BCAAs) activate the mTOR signaling pathways, critical in muscle protein and RNA synthesis regulation [22].

Similarly, arginine, glutamine, and cysteine have been shown to facilitate and promote skeletal muscle growth and development [23–25]. In addition, amino acids also play an essential role in regulating various signaling pathways involved in skeletal muscle metabolism, such as the insulin signaling pathway, which is responsible for glucose uptake and energy metabolism in skeletal muscle cells [26]. Overall, amino acids are crucial for skeletal muscle growth and development by activating various signaling pathways involved in muscle metabolism, growth, or performance [27].

In this study, we first explore different amino acid types and contents in HME and BME by targeted metabolomics. Second, we would evaluate myogenic effects and find relative mechanisms for the HME and BME impacting C2C12 cells and immature mouse quadriceps by regional intramuscular injection, avoiding the first-pass effect, respectively. Meantime, we also used targeted metabolomics to further analyze the amino acid types and contents in skeletal muscle after HME or BME treatment linked with muscle growth and performance by Pearson correlation analysis. Finally, we hope our study can provide novel insights and potential ideas about food additives, drug carriers or other milk functions in future research.

## Materials and methods

### Milk samples collection

**Bovine milk collection:** We obtained unpasteurized milk from healthy Hustin dairy cows within 60 days after giving birth at the Beixing farm (Heilongjiang province, China). Cold chain transportation immediately transported the milk tanks to the sample library [9].

**Human milk collection**: From October 2021 to May 2022, we recruited 10 lactating mothers at the Department of Obstetrics and Gynecology, Tongji Hospital, Tongji medical college, Huazhong University of Science and Technology, China. All mothers had healthy, full-term vaginal births and were 29 ± 1.58 years old without other diseases. All mothers also produced an excess amount of milk for their infants. Before participating in the study, the donors were informed of the purpose of the research and provided informed consent. Meantime, considering reality, feasibility, and some authority guidelines, when they received care in the first 3 weeks postpartum in the clinical department [28–31], we collected 200 mL of mature milk from each mother (Donors briefly information: **Table**S1), which were immediately saved at 4 °C and kept in this state during transportation to the sample library. The research was approved by the Medical Ethics Committee of Tongji Medical College of Huazhong University of Science and Technology (IRB Number: S237) and registered at the China Clinical Test Registration Center (Registration Number: ChicTR2100052827). To investigate the effects of HMEs or BMEs on skeletal muscle, we mixed freshly collected human milk or bovine milk samples in equal volumes, ensuring similarity among the volunteers’ backgrounds.

### Preparation of EVs by differential ultracentrifugation

HME and BME were isolated from milk samples using differential centrifugation, referring to established protocols [32, 33]. Briefly, the milk sample was initially centrifuged at 13,000 × g for 30 min at 4 °C (Allegra 64R, Beckman Coulter, United States), and the resulting supernatant was collected to remove fats, cells, and debris. The supernatant was centrifuged at 100,000 × g for 60 min at 4 °C using a SW41Ti rotor and an ultracentrifuge (Optima XE-100, Beckman Coulter, United States). The lower slush portion, along with the pellet, was discarded to eliminate larger particles and micro-vesicles. Finally, the remaining supernatant was centrifuged at 145,000 × g for 90 min at 4 °C to discard it, and the EVs pellet obtained was re-suspended in phosphate-buffered saline (PBS, 0.01 M) and subjected to another round of ultracentrifugation and washing. After removing residual sediment through centrifugation at 10,000 × g for 5 min at 4 °C, the resulting EVs suspension was sterilized via filtration with a 0.22 μm filter. The total protein content of EVs was quantified, adjusted to 6 mg/mL, divided into equal aliquots, and stored at -80 °C until use (avoiding repeated freezing and thawing).

### EVs confirmation

#### Transmission electron microscopy (TEM)

The profile or morphology of HME and BME were examined using TEM (FEI Tecnai 12, Philips, Holland). Briefly, 10 µL of the sample was pipetted dropwise onto a copper grid and allowed to settle for 1 min. The suspension was then removed with filter paper before adding 10 µL of uranyl acetate dropwise onto the copper mesh for another minute. The grid was dried at room temperature for several minutes before imaging with TEM at 100 kV to visualize the EVs.

#### Nanoparticle tracking analysis

HME and BME samples were pre-diluted 1:100 in phosphate-buffered saline (PBS, 0.01 M) and loaded into a 1 mL syringe. The measurements were carried out using a nanoparticle tracking analyzer (NTA; NanoSight NS300, Malvern, UK), during which the samples were analyzed under scattered light signals while being pumped at a syringe pump speed of 75 µL/min. Three 60-second videos were recorded, with 30 frames per second, and analyzed using the NTA 3.3 Dev Build 3.3.104 software. This software tracked and analyzed the Brownian motion of each particle to determine their hydrodynamic radius and concentration.

#### Western blotting for EVs protein markers

To detect EVs protein markers CD81 and TSG101 and all procedures referred to previous publications [34], we loaded 20 µg of protein samples onto a 12% SDS-PAGE gel and separated them. Subsequently, we transferred the proteins onto a 0.45 μm nitrocellulose (NC) membrane (Millipore, HATF00010, USA) and blocked it for an hour using 5% nonfat milk in TBST. The membrane was then incubated overnight at 4 °C with a primary antibody at a 1:1000 dilution. Primary antibodies of CD81 (ab109201) and TSG101 (ab133586) were obtained from Abcam (Cambridge, UK). We incubated the membrane with a horseradish peroxidase-conjugated secondary antibody to detect the proteins for an hour. Finally, we analyzed the images collected by GeneSnap (Syngene, Cambridge, UK) and GeneTools (Syngene, GeneTools 4.0, Cambridge, UK) after putting the NC membrane sprinkled with ECL coloring solution into the gel imaging analyzer (Syngene, UK).

### Cell lines and treatments

#### Cell culture conditions

C2C12 cells were obtained from The American Type Culture Collection (ATCC, Manassas, USA), and cells were cultured in 6-well plates containing 3 mL of the medium at a density of 3 × 10^5^ cells per well under 5% CO2 and 37 °C. The growth medium comprised 10% (v/v) fetal bovine serum (GIBCO), 2 mm glutamine (GIBCO), 100 U/mL penicillin, and 100 µg/mL Dulbecco’s modified Eagle’s medium (DMEM; GIBCO). After the cells reached 70–80% confluence, the medium was replaced with a differentiation medium consisting of 2% (v/v) horse serum (GIBCO), 2 mm glutamine, 100 U/mL penicillin, and 100 µg/mL DMEM. The differentiation medium was refreshed every 24 h for 7 days. Myotube formation was monitored daily using an inverted microscope.

#### BME and HME treatments

Following the differentiation of C2C12 cells, we added 5 × 10^8^ EVs to each well of a 6-well plate and incubated the cells for 24 h in an EVs-Free medium [35]. After the incubation period, we collected the cells and re-suspended them in PBS containing a protease inhibitor cocktail (B1400) and phosphatase inhibitor cocktail (B15001) from Bimake (Houston, USA). The cells were placed on ice and sonicated using an amplitude of 30% for 10 s, with this process repeated three times. We collected the cell supernatant via centrifugation at 3000 rpm for 10 min and quantified the total protein in the supernatant using the BCA method. The supernatant was stored at -20 °C for subsequent protein blot analysis.

#### C2C12 cells uptake EVs

In our study, we employed the red fluorescent dye DiI (1,1-dioctadecyl-3,3,3,3- tetramethylindocarbocyanine perchlorate, G1705, Servicebio, Wuhan, China) to label EVs, the green fluorescent dye DiO (3,3’-dioctadecyloxacarbocyanine perchlorate, G1704, Servicebio, Wuhan, China) to visualize C2C12 cell membranes, and the blue fluorescent dye Hoechst-33,342 (G1127, Servicebio, Wuhan, China) to mark cell nuclei. These dyes allow for efficient and accurate tracking of the cellular uptake of EVs. Specifically, BME or HME was labeled with a 1:1000 working solution of DiI, and cell membranes were labeled using a 1:1000 working solution of DiO, and cell nuclei were labeled using a 10 µg/mL Hoechst 33,342 working solution, respectively. Excess fluorescent dyes were washed away after incubating at 37 °C for 30 min in the dark. To investigate cellular uptake, labeled BME or HME particles (5 × 10^8^) were added to each well containing C2C12 cells in a 6-well plate, and their internalization was examined by inverted fluorescence microscopy. Meanwhile, all experimental procedures were carried out according to the manufacturer’s instructions.

#### Jenner-Giemsa staining and figure analysis

In this study, we employed Jenner-Giemsa staining to analyze myotubes in vitro. First, C2C12 cells were washed with PBS, fixed with 100% methanol for 5 min and dried at room temperature for 10 min. Subsequently, cells were incubated for 5 min with diluted Jenner dye in distilled water, followed by 5 minutes’ exposure to Giemsa dye diluted in 1:5 distilled water. After washing with distilled water, myotubes were visualized using an inverted microscope and analyzed with Image J (Version 1.52i, National Institutes of Health, Bethesda, MD, United States). We quantified muscle fibers of around 30 myotubes per well (total 180 myotubes per picture).

According to the previous research [36], the myotubes were analyzed in an unbiased manner after being dyed with Jenner-Giemsa. The RGB images were converted into gray data using Image J, which were then presented in gray tones. The image histogram analysis was obtained by selecting “Analysis/Reduction”. The X-axis of the image represents the range of 255 Gy tones (0 = black, 255 = white), while the Y-axis represents the pixel number of each color. For each image, the histogram of the myotubes exhibited a large proportion of pixels attributed to the darkest tone. The average pixels attributed to each image were calculated for the average pixel of the tone 0-X between each condition to quantify the density of the myotubes. The darkest threshold (X) was arbitrarily selected, but it depended on the shape of the histogram. The shape may vary from experiment to experiment and should include the most different tone intervals between conditions. It is essential to note that all the images in a single experiment must be used with the same threshold.

#### Cell viability assay

According to the manufacturer’s protocol, the cell viability assay was performed with Cell Counting Kit-8 (CCK-8; Dojindo, Japan). C2C12 cells were seeded in a 96-well plate at a density of 1 × 10^4^ cells per well. Before assessing cell viability using the CCK-8 assay, cells were treated with different concentrations of Rapamycin (3.125, 6.25, 12.5, 25, 50, and 100 nM) for 24 h. Subsequently, the culture medium in the 96-well plate was replaced with 100 µL of culture medium containing CCK-8 and incubated at 37 °C for 1 h. The absorbance at 450 nm was measured using a microplate reader. Cell viability was expressed as a percentage relative to the control (untreated) cells.

### Animals care, treatment, and study design

#### Animals’ housing and treatment

Male C57BL/6J mice, aged 21 days (3 weeks), were acquired from Life River Experimental Animal Technology Company (Beijing, China). The mice were housed in a specific pathogen-free animal laboratory, maintained under a 12-hour light/dark cycle with regulated ventilation (air exchange rate of 18 times per hour). They provided ad libitum access to food and water. The Institutional Animal Care and Use Committee (IACUC Number: 3030) of Tongji Medical College of Huazhong University of Science and Technology approved the experimental protocol. It was conducted following the guidelines for experimental animal care and use established by the National Health Research Institute.

#### Experiment design

Following adaptive feeding for one week, mice were randomly divided into three groups (weighing: 15.525 ± 0.709 g): a control group, an HME group and a BME group (N = 12/group). The HME and BME groups received injections of HME and BME into both their quadriceps. To ensure consistent dosing, we injected 80 µL of extracellular vesicles suspension (equivalent to 5 × 10^9^ vesicles/40 µL per quadriceps injection site) into the bilateral quadriceps of the mice every three days for a total of 30 consecutive days [35]. The control group received PBS injections following the same methods. After the experiment, we euthanized the mice by cervical dislocation and dissected them immediately. We collected the quadriceps and stored them at -80 °C for subsequent index determination.

#### H&E staining

The quadriceps were fixed in 4% paraformaldehyde for at least 24 h, embedded in paraffin, and sliced into 4-µm sections using a Leica microtome (Solms, Germany). H&E staining was applied for morphological analysis. The Olympus IX-71 microscope (Tokyo, Japan) observed the quadriceps sections.

#### Muscle fibers cross-sectional area (CSA) measurements

According to our published study [37], six different regions of the quadriceps muscle were imaged at 200× magnification using H&E-stained sections. For each image, 50 consecutive muscle fibers were manually traced to obtain an average of 300 fibers per muscle. Muscle fibers CSA values were calculated using Image J software (Version 1.52i, National Institutes of Health, Bethesda, MD, USA) by determining the area and number of traced fibers.

#### Collagen analysis

Paraffin-embedded tissue sections were deparaffinized and hydrated on glass slides. Subsequently, the slides were immersed in a solution of Sirius Red dye for 1 h, followed by a 30-second rinsing with running water and dehydration using ethanol. After clearing the slides with a xylene-based clearing agent, they were mounted and images were captured. The Image-Pro Plus software was utilized to calculate the area fraction occupied by collagen fibers. Additionally, a polarizing microscope (Eclipse Ci-L, Nikon, Japan) was employed to examine the slides and visualize collagen birefringence. Collagen I was identified as red to orange fibers, while collagen III was identified as green fibers.

### Grip test

Grip strength was measured using a custom apparatus designed and manufactured by Yiyang Technology Development Co., Ltd. (XR-YLS-13 A, Jinan, China). Mice were gently held by the tail and allowed to grasp the metal wire mesh of the grip strength meter. According to a relative study, the mice were then gently pulled back until they released the mesh. During the first week, second and a half week, and fourth week of BME and HME intervention periods, grip strength was measured in each mouse six times [38].

### Coordination-fatigue test: rotarod test

According to published research [39], we employed a rotary fatigue instrument (XR-6 C, Xinruan information and technology Ltd, Shanghai, China) to train and test small animals. The “Overall rod performance” (ORP) was used to determine the area under the rotor’s time and rotation speed curve, thereby scoring and summarizing the overall performance of the test subjects. During the initial week of the experiment, the small animals were trained and familiarized with rotary skills and instruments. Subsequently, ORP was measured on the second and fourth week of the BME and HME intervention period, with each mouse receiving three repeated measurements.

### Biochemical index measurement for serum samples

Mouse serum samples were placed at 4 °C and centrifuged at 3000 rpm for 15 min. Then, to assess the impact of HME and BME on the physiological functions of animals, we employed a fully automated biochemical instrument (Shenzhen Read Life Technology, Shenzhen, China) to measure levels of creatine kinase, uric acid, urea nitrogen, total protein, calcium, alanine aminotransferase, and aspartate aminotransferase in the serum.

### Western blotting

To extract total protein from C2C12 cells and mouse quadriceps, we loaded 40 µg of protein samples onto a 10% SDS-PAGE gel and transferred the protein to a nitrate cellulose membrane. Our experimental methods are referred to published study [40]. We used primary antibodies against AMPKα (#5831, 1:1000, Cell Signaling Technology, MA, USA), p-AMPKα (thr172) (#2535, 1:1000, Cell Signaling Technology, MA, USA), SIRT1 (#9475, 1:1000, Cell Signaling Technology, MA, USA), PGC-lα (ab54481, 1:1000, Abcam, UK), AKT (#4691, 1:1000, Cell Signaling Technology, MA, USA), p-AKT (#4060, 1:1000, Cell Signaling Technology, MA, USA), mTOR (#2983, 1:1000, Cell Signaling Technology, MA, USA), p-mTOR (#5536, 1:1000, Cell Signaling Technology, MA, USA), p70S6 Kinase (#34,475, 1:1000, Cell Signaling Technology, MA, USA), Phospho-p70S6 Kinase (#9205, 1:1000, Cell Signaling Technology, MA, USA), 4EBP1 (#9644, 1:1000, Cell Signaling Technology, MA, USA), p-4EBP1 (#2855, 1:1000, Cell Signaling Technology, MA, USA), Myog (ab1835, 1:250, Abcam, UK, observed band size in this study: 55KD), Myf5 (ab125301, 1:1000, Abcam, UK), Myod1 (A16218, 1:1000, ABclonal, Wuhan, China), and GAPDH (#5174, 1:10000, Cell Signaling Technology, MA, USA). We used secondary HRP-linked antibodies (#7076, 1:10000, Cell Signaling Technology, MA, USA) and Lumigen ECL Ultra (Lumigen, MI, USA) detection reagents to visualize the proteins.

### Targeted metabolomics analysis for HME, BME and quadriceps

In this section, previous publications referred to our sample preparation, extraction, and LC-ESI-MS/MS system conditions [41, 42].

#### HME and BME samples preparation and extraction

To prepare the sample for targeted metabolomics analysis of amino acid and its’ metabolites, the frozen sample was thawed on ice and 0.4 mL of it was freeze-dried. The freeze-dried sample was then mixed with 500 µL of 80% methanol/water pre-cooled at -20 °C. The mixture was vortexed for 2 min at 2500 r/min. The sample was then frozen in liquid nitrogen for 5 min and left on ice for 5 min before being vortexed again for 2 min. This step was repeated three times. The sample was then centrifuged at 12,000 r/min for 10 min at 4 °C. After centrifugation, 300 µL of the supernatant was taken and sonicated at -20 °C for 30 min. The supernatant was then centrifuged at 12,000 r/min for 10 min at 4 °C. Finally, 200 µL of the supernatant was transferred through a Protein Precipitation Plate for further LC-MS analysis. This method ensures the efficient preparation of the sample for LC-MS analysis while minimizing the risk of contamination or loss of analytes.

#### Quadriceps samples preparation and extraction

After thawing and homogenizing the muscle samples, 0.05 g was mixed with 500 µL of 70% methanol/water and vortexed for 3 min at 2500 r/min. The mixture was then centrifuged at 12,000 r/min for 10 min at 4 °C. The supernatant (300 µL) was transferred to a new centrifuge tube and stored at -20 °C for 30 min before being centrifuged at 12,000 r/min for 10 min at 4 °C. Finally, 200 µL of supernatant was transferred through a protein precipitation plate for LC-MS analysis.

#### UPLC Conditions

An LC-ESI-MS/MS system (UPLC, ExionLC AD, https://sciex.com.cn/; MS, QTRAP® 6500 + System, https://sciex.com/) was used to analyze the sample extracts. The HPLC column used was ACQUITY BEH Amide (i.d.2.1 × 100 mm, 1.7 μm) and the solvent system consisted of water with 2 mM ammonium acetate and 0.04% formic acid (A) and acetonitrile with 2 mM ammonium acetate and 0.04% formic acid (B). The gradient was initiated at 90% B (0-1.2 min), decreased to 60% B (9 min), 40% B (10–11 min) and finally ramped back to 90% B (11.01-15 min). The flow rate was set at 0.4 mL/min and the temperature was maintained at 40 °C. The injection volume used was 2 µL.

#### ESI-MS/MS conditions

The AB 6500 + QTRAP® LC-MS/MS System has an ESI Turbo Ion-Spray interface and can operate in positive or negative ion modes. It is controlled by Analyst 1.6 software (AB Sciex). The ion source is a turbo spray with a source temperature of 550 °C. The ion spray voltage is set to 5500 V for positive mode and − 4500 V for negative mode. Curtain gas is set to 35.0 psi. We optimized DP and CE for each MRM transition. We monitored a specific set of MRM transitions for each period based on the amino acid eluted within that period.

### Statistical analysis

The preprocessed amino acids and their metabolites dataset was imported into Metabo-Analyst for principal component analysis (PCA), orthogonal partial least squares discriminant analysis (OPLS-DA), and volcano plot analysis. Metabolite species that differed significantly between groups were screened using the criteria of variable importance in projection (VIP) > 1, p < 0.05, and fold change (FC) > 2 (up-regulated) or FC < 0.5 (down-regulated). Pathway enrichment analysis and metabolite annotation were performed using the Kyoto Encyclopedia of Genes and Genomes (KEGG) metabolic pathways database. Statistical analysis and data processing were conducted using Prism software (GraphPad Prism 8.0). All results are presented as mean ± standard error (x ± SE), and all data were generated from at least three independent experiments. Multiple groups were compared using one-way analysis of variance (ANOVA), and significance was defined as p < 0.05. More details for “Targeted metabolomics analysis” showed in “Materials and methods S1”.

## Results

### Biological characteristics of HME and BME

**Fig. 1:**
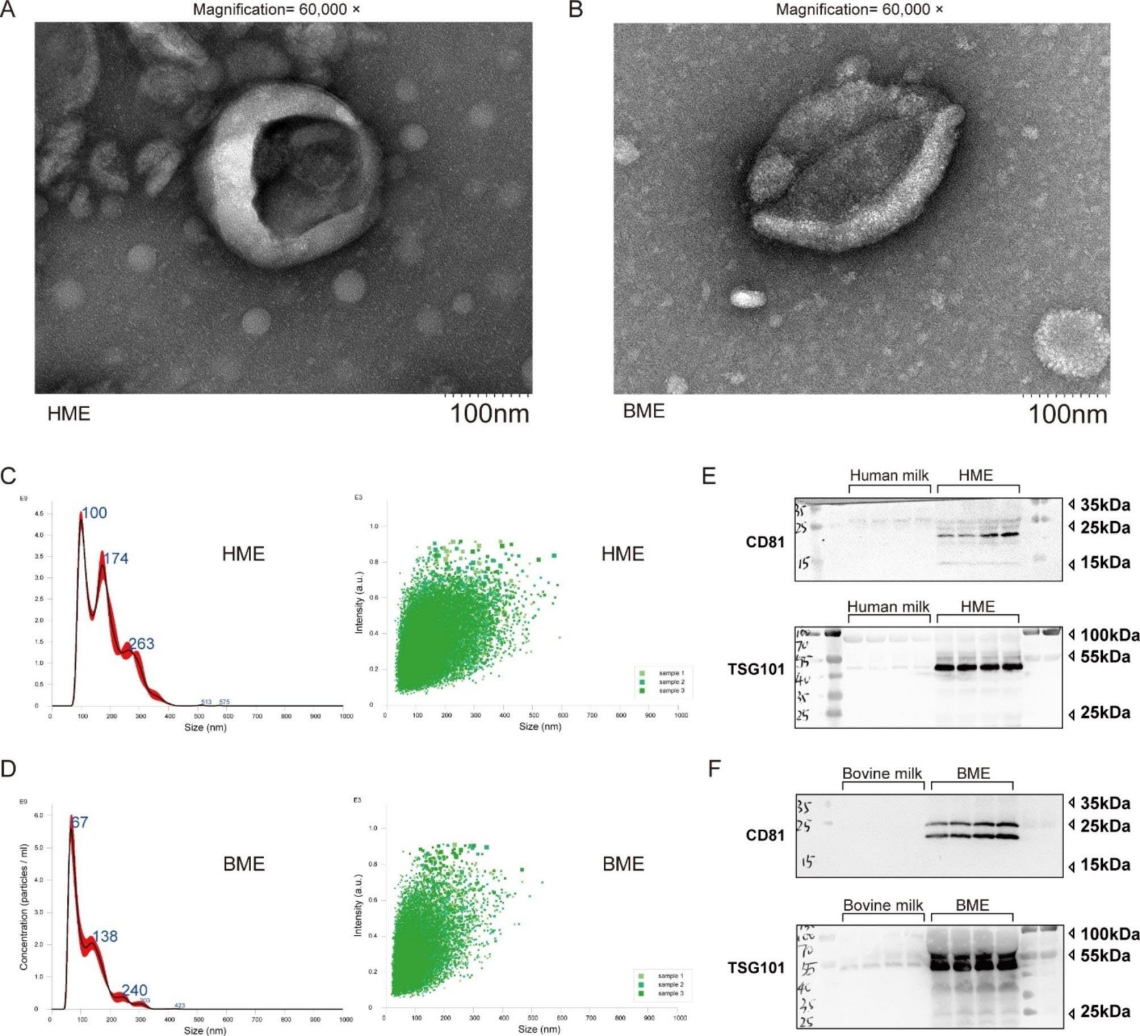
Characterizations of HME and BME. (**A**-**B**): Representative figures for the morphology of HME and BME by Transmission electron microscopy (TEM). (**C**-**D**): Figures for Nanoparticle tracking analysis (NTA) analyzed particle size of HME and BME. (E-F) Typical pictures for extracellular vesicle markers by Western blotting detection

The HME and BME were comprehensively characterized using various techniques, including TEM, NTA and Western-blotting, respectively. Figures from TEM illustrated that both HME and BME exhibited as similar as “cup-shaped morphology” (Fig. 1A-B **and Fig.**S1-S2). Besides, the average particle sizes for HME, as measured by NTA, were 179.2 ± 1.5 nm, whereas for BME, sizes were 118.6 ± 0.4 nm (Fig. 1C-D **and Table**S2). Pictures from Western blotting also revealed that EVs-specific markers TSG101 and CD81 were highly enriched in both HME and BME samples, demonstrating highly purified EVs. Conversely, TSG101 and CD81 were either not detected or only present in low concentrations in milk serum (Fig. 1E-F).

### Comprehensive analysis of the amino acid spectra or profile of HME and BME by targeted metabolomics

#### Sample quality control and data analysis

We utilized UHPLC-MS/MS to detect amino acids in HME and BME. Our method demonstrated high sensitivity, allowing for the simultaneous detection and quantification of various amino acids and their metabolites. Notably, the UHPLC system effectively separated and eluted different amino acids and metabolites from HME and BME within 12 min (**Fig.**S3A). A database based on the Metware Database (MWDB, Metware Bio-technology Ltd, Wuhan, China) was established, which allowed for qualitative analysis of mass spectrometry data, and quantitative analysis was performed using the multiple reaction monitoring (MRM) modes of triple quadrupole mass spectrometry. Using standard curves, chromatographic peaks of all target compounds were integrated for quantitation analysis after obtaining the spectral analysis data of different samples. There was a high degree of stability in the experimental data, as evidenced by highly overlapping TIC charts for multiple QC samples, a Pearson correlation coefficient R2 close to 1 between QC samples, and more than 80% of compounds with CV values less than 0.2 (**Fig.**S3A-C).

#### Amino acid and metabolites profiles of HME and BME using clustering and visualization methods

**Fig. 2:**
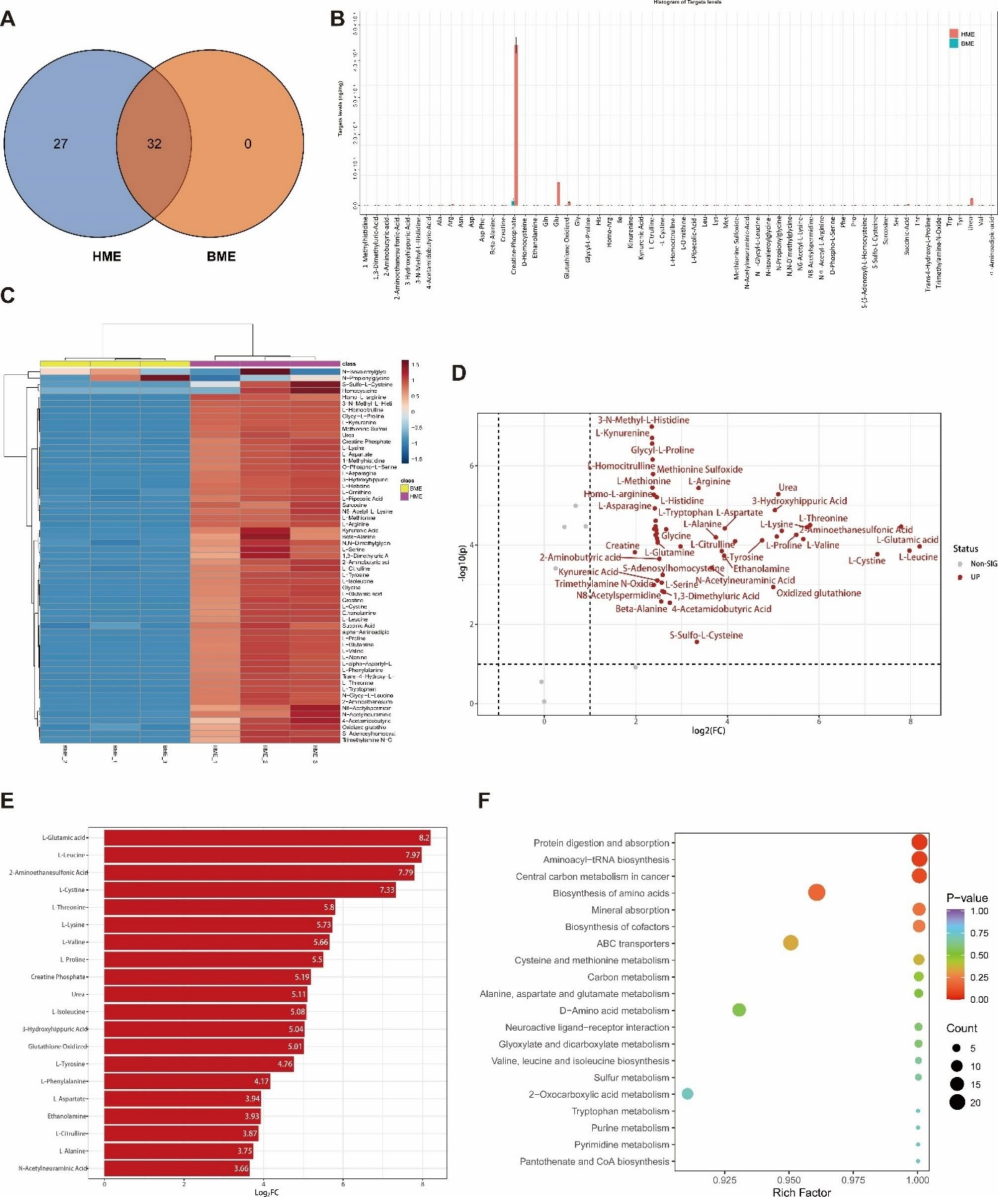
Figures for amino acid profiling in HME and BME samples by targeted metabolomics analysis. (**A**): Venn diagram of amino acid composition for HME and BME. (**B**): Bar chart showing the content of amino acids and their metabolites in HME and BME. (**C**): Relative abundance heat map of amino acids and their metabolites in HME and BME. (**D**): Volcano plot analysis for significantly different metabolites between HME and BME. (**E**): Top 20-fold change metabolites. (**F**): KEGG enrichment analysis for different metabolites

In total, we detected 59 amino acids and their metabolites in HME samples, while 32 were identified in BME. However, all amino acids detected in BME were also present in HME (Fig. 2A). Furthermore, PCA and OPLS-DA analyses revealed separation trends in amino acid components between HME and BME (**Fig. S4A-B**). According to the metabolic characteristics of six milk-derived extracellular vesicle samples, two categories of EVs were identified based on their milk sources HME and BME, through partitional clustering and hierarchical clustering (**Fig. S4C-D)**. To provide a more intuitive illustration of the substance content differences between the HME and BME, bar graphs and a table for amino acid and metabolite contents of HME and BME were plotted. The results showed that Creatine-Phosphate levels were much higher in HME than other metabolites. Creatine-Phosphate is a high-energy phosphate compound found in muscle cells responsible for releasing high-energy phosphate groups that help produce ATP by reacting with ADP. This provides a rapid energy source for high-intensity and short-duration activities and is essential for skeletal muscle contraction and movement (Fig. 2B **and Table**S3). Moreover, heat maps of the relative distribution of amino acids and their metabolites revealed that nearly all these compounds were higher in HME than in BME (Fig. 2C).

#### Differential metabolites screening using volcano plot, z-score analysis and random forest models analysis

Volcano plot analysis to screen metabolites that showed significant differences between HME and BME, metabolites with *p* < 0.05, fold change (FC) > 2 (up-regulated), or FC < 0.5 (down-regulated) were selected as important features. Our results showed that 52 amino acids or their metabolites were significantly higher in HME than in BME, and we displayed the top 20 substances with the highest differential multiples (Fig. 2D-E). We then normalized the differential metabolites in HME and BME using Z-scores analysis to observe their distribution of HME and BME. The differential metabolites in BME had a Z-score less than 0, whereas those in HME had a Z-score greater than 0 (**Fig. S5A**). Further, we constructed random forest models analysis for amino acids and their metabolites in HME and BME, with mean decrease accuracy showing the degree of decrease in random forest prediction accuracy. A large value indicated that the variable was more important, and we found that glycine was the most significant variable between HME and BME. Besides, glycine is the basic unit of muscle protein and is essential for synthesizing muscle fibers. It is also known to be associated with skeletal muscle health and function, with studies showing that supplementing with glycine can increase endurance and strength in athletes while promoting muscle protein synthesis. Additionally, glycine is an antioxidant that can reduce oxidative damage to muscles (**Fig. S5B**).

#### KEGG annotation and enrichment analysis

The predictive pathways identified in this study were annotated and classified by the Kyoto Encyclopedia of Genes and Genomes (KEGG, Japan), revealing the metabolic pathways (ko01100) to be the most highly annotated KEGG pathway (**Fig. S6A**). Further, KEGG enrichment analysis was performed, along with an analysis of the overall changes in KEGG metabolic pathways. The differential metabolites identified in HME and BME were significantly enriched in the protein digestion and absorption pathway (ko04974) and the aminoacyl-tRNA biosynthesis pathway (ko00970). In skeletal muscles, the amino acids produced during protein digestion and absorption are utilized to synthesize muscle proteins and participate in various biological processes such as muscle metabolism and cell signaling. Aminoacyl-tRNA synthesis is a crucial step in the protein synthesis process, in which amino acids are combined with corresponding tRNA molecules to form aminoacyl-tRNA necessary for ribosomes to perform the translation and synthesize specific proteins. In skeletal muscles, aminoacyl-tRNA synthesis is important for the continuous synthesis and repair of muscle proteins and hence plays a critical role in skeletal muscle health and function (Fig. 2F **and Fig. S6B**).

### HME triggers C2C12 myotubes in densities, lengths, as well as expression of AKT-mTOR-p70s6k pathway increasing

**Fig. 3:**
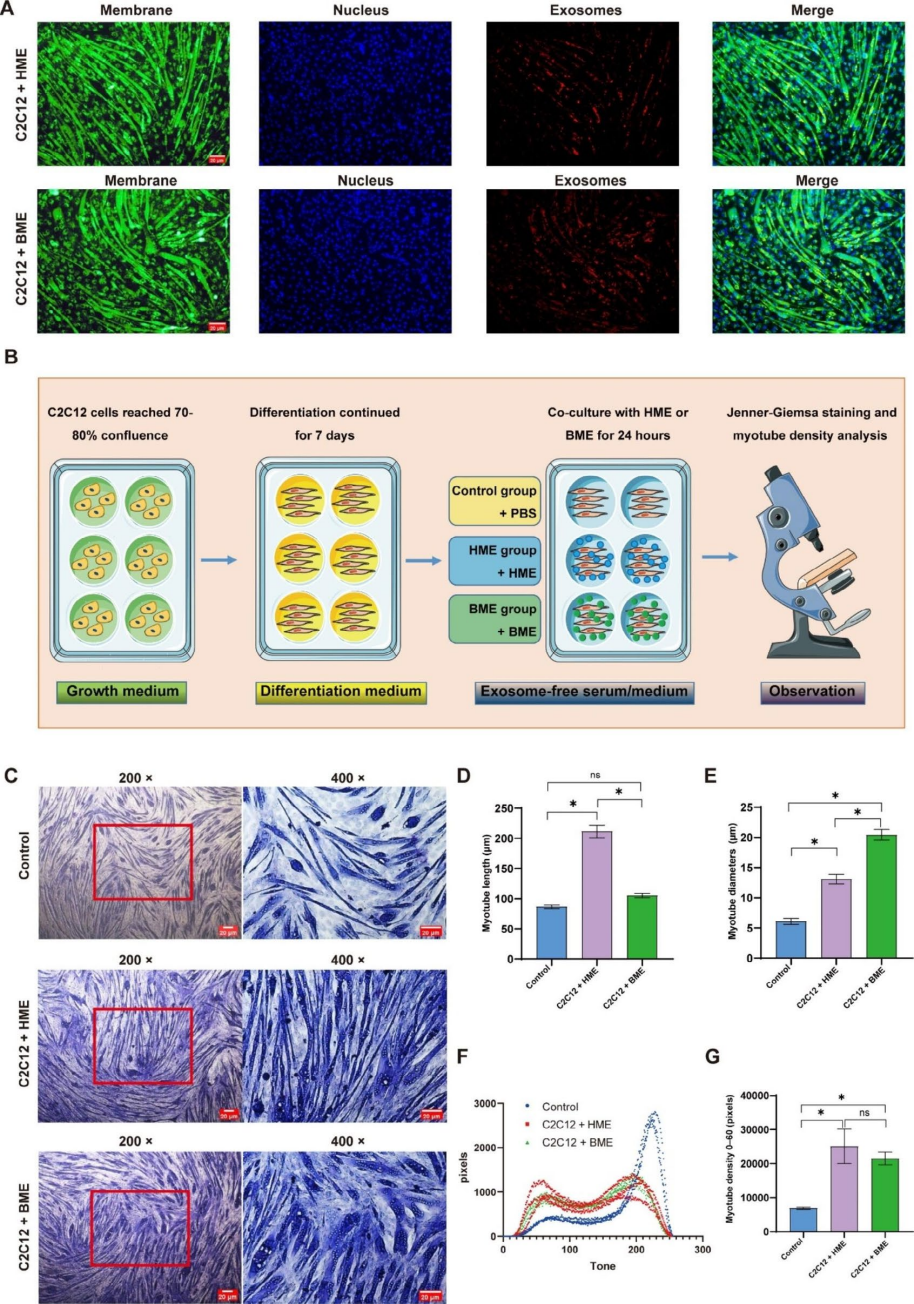
The figures for study design, HME and BME up-taken by C2C12cells, and effects of HME and BME on C2C12 cells (myotubes) differentiation. (**A**): The figures for HME/BME co-cultured with C2C12 cells, 3,3’-dioctadecyloxacarbocyanine perchlorate (DiO)-stained myotubes emit green fluorescence, Hoechst-stained nuclei emit blue fluorescence, and 1,1’-dioctadecyl-3,3,3’,3’-tetramethylindocarbocyanine perchlorate (DiI)-stained EVs emit orange-red fluorescence. (**B**): Experimental design for cell culture. (**C**): Representative Jenner-Giemsa staining images of myotubes. (**D**-**E**): Effects of HME and BME on the length and diameter of C2C12 myotubes. (**F**-**G**): Myotubes densities are calculated as the sum of pixels attributed to tones 0–60. Data are presented as mean ± SE, the * indicates *p* < 0.05

To investigate the impact of HME and BME on myotubes differentiation, it was necessary first to determine their uptake by C2C12 cells. After co-culturing DiI-stained HME and BME with C2C12 cells for 3 h, pictures from fluorescence microscopy revealed their even localization within the C2C12 cells (Fig. 3A). Next, Jenner-Giemsa staining was employed on C2C12 cells that were co-cultured with HME and BME for 24 h to analyze the length and diameter of myotubes (Fig. 3B-C). Additionally, we calculated the distribution of pixels in the grey tone range to quantify C2C12 differentiation, which provided a simple and unbiased measurement of myotube densities. Significantly, our results also demonstrated that treatment with HME significantly increased myotubes length and diameter compared to the control group. Similarly, BME treatment significantly up-regulated myotubes diameter compared to the control group (Fig. 3D-E). Importantly, both HME and BME treatments substantially increased myotubes densities (Fig. 3F-G).

**Fig. 4:**
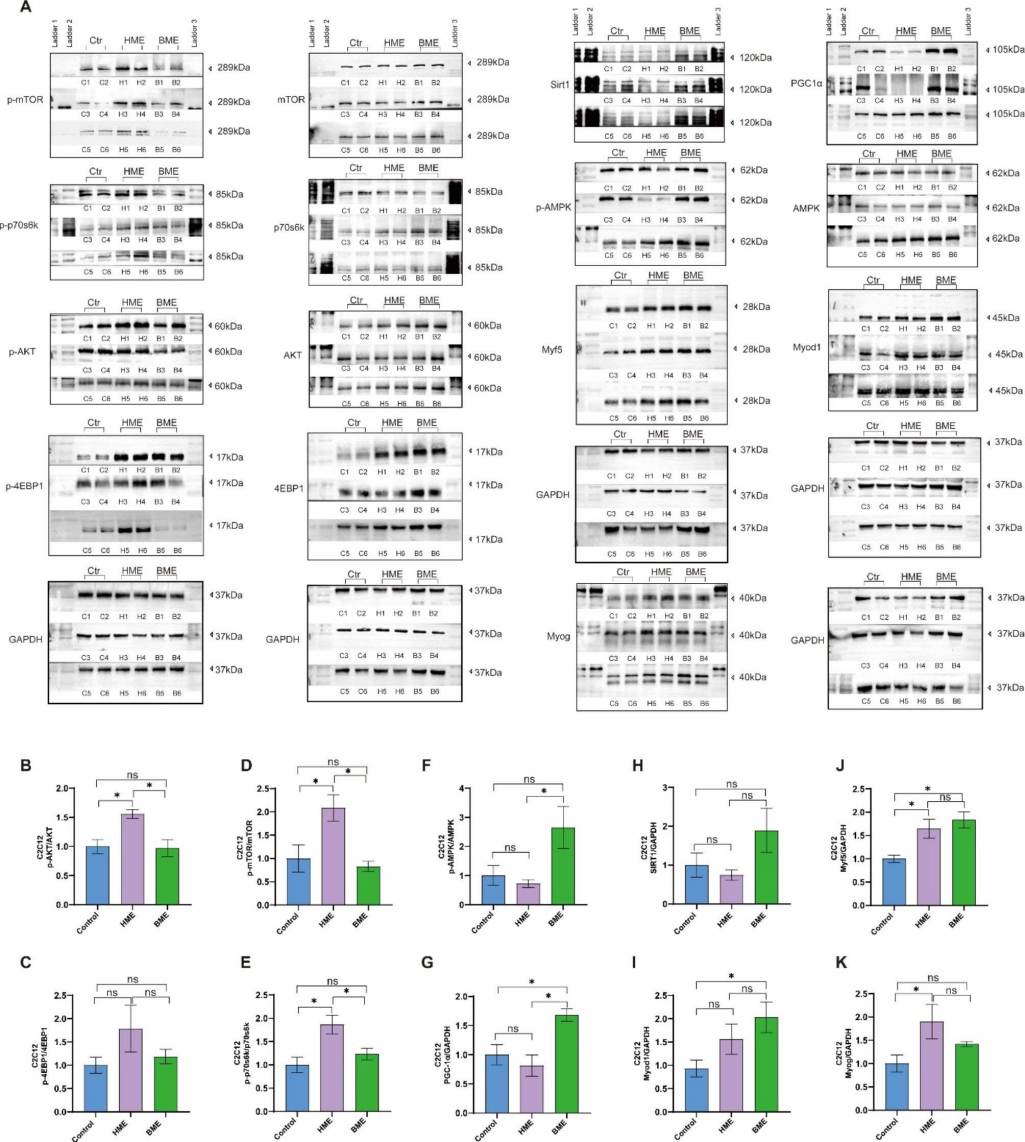
Effects of HME and BME on AKT-mTOR-P70S6K-4EBP1 pathway, AMPK-SIRT1-PGC-1α pathway and regulation of myogenic regulatory factors in C2C12 cells. (**A**): Figures for Western blotting detection, where C1-C6 lanes for control group samples, H1-H6 lanes for samples intervened by HME, and B1-B6 lanes for samples intervened by BME. (**B**-**E**): Protein levels of p-Akt/Akt, p-mTOR/mTOR, p-P70S6K/P70S6K, and p-4E-BP1/4E-BP1 in the C2C12 cells. (**F**-**H**): Protein levels of p-AMPK/AMPK, SIRT1, and PGC-1α. (I-K): Protein levels of myogenic regulatory factors Myog, Myf5, and Myod1 in the C2C12 cells. Data are presented as mean ± SE, the * indicates *p* < 0.05

Then, we also investigated and testified the potential signaling pathways of HME and BME effecting C2C12 cells according to KEGG analysis. The AMPK-SIRT1-PGC-1α pathway, which governs energy homeostasis in skeletal muscle [43], was analyzed alongside the AKT-mTOR- p70s6k -4EBP1 pathway, which is central to muscle growth, metabolism, and protein synthesis [44]. Muscle Regulatory Factors (MRFs), a group of transcription factors like Myf5, MyoD, and Myogenin, are essential to skeletal muscle development, regulating myoblast differentiation, maturation, and growth into myofibers. Besides, MRFs play a direct or indirect role in skeletal muscle protein synthesis, further regulating muscle tissue growth and function. Following a 24-hour treatment with HME, up-regulation patterns of p-AKT/AKT, p-mTOR/mTOR, p-p70s6k/p70s6k, Myf5, and Myog were observed after HME treatment compared to the control. Notably, HME treatment did not affect the expression of p-AMPKα/AMPKα, SIRT1, and PGC-1α (Fig. 4A-K). Therefore, these findings suggest that HME can enhance MRFs activity, promoting muscle regeneration, differentiation and increasing muscle mass and function. Conversely, after 24 h of BME treatment, we observed increased expression levels of PGC-1α, Myf5, and Myod1, but no significant effect was observed on the AKT-mTOR-p70s6k-4EBP1 pathway (Fig. 4A-K). PGC-1α is a transcriptional coactivator capable of mitochondrial biogenesis stimulation and regulating skeletal muscle energy homeostasis while promoting metabolic balance. Although BME did not alter the expression of the AKT-mTOR-p70s6k-4EBP1 pathway, it caused a significant increase in the diameter and density of myotubes, along with the activation of MRFs. Consequently, it is possible that BME transiently activates the mTOR signaling pathway early on.

**Fig. 5:**
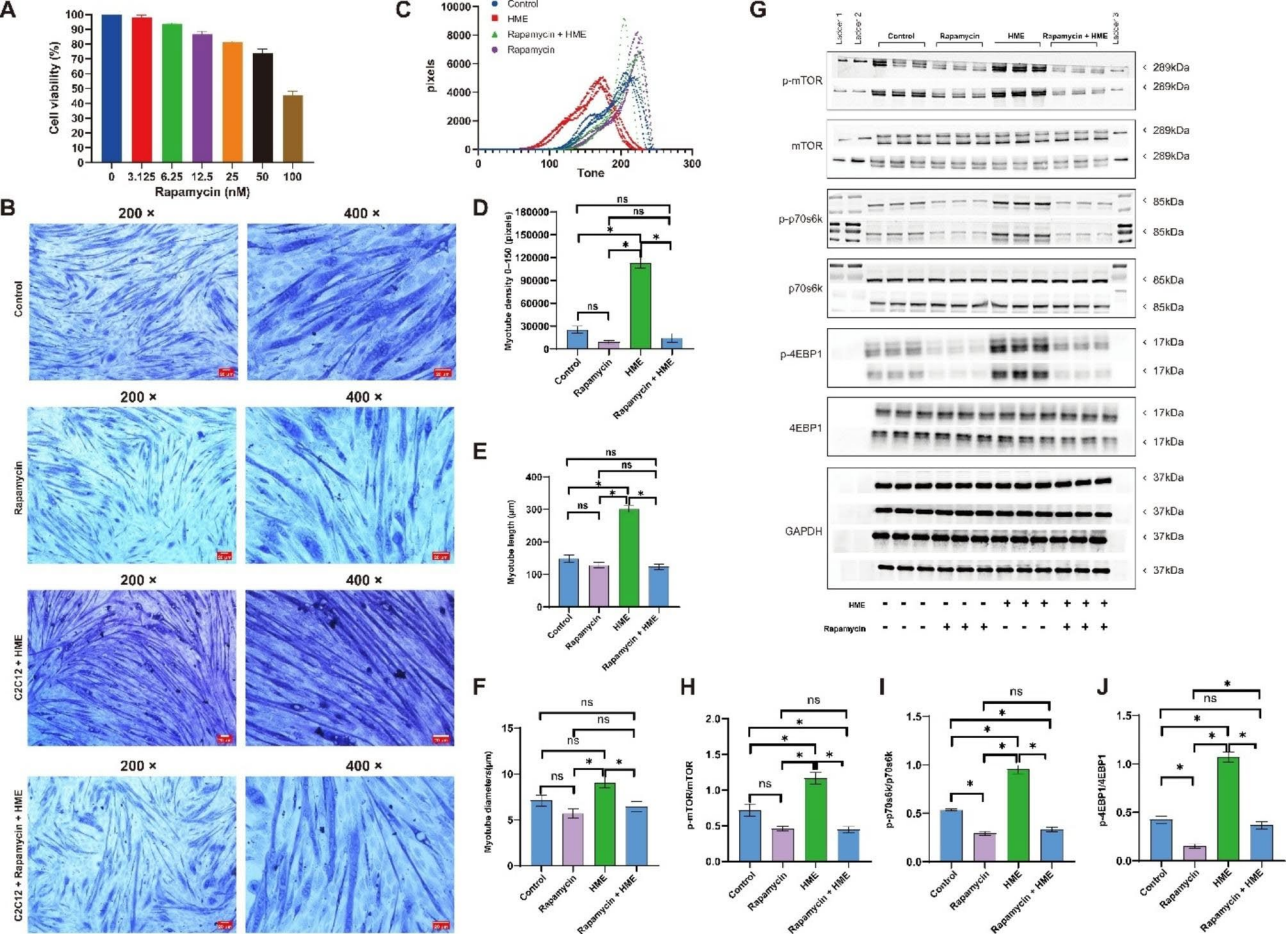
Combining HME with Rapamycin at the C2C12 level. (**A**): Rapamycin exerts an inhibitory effect on C2C12 cells in a dose-dependent manner, the cell survival rate of the control group was set to 100%. (**B**): Representative Jenner-Giemsa staining images of myotubes. (**C**-**D**): Myotubes densities calculated as the sum of pixels attributed to tones 0-150. (**E**-**F**): Effects of HME and BME on the length and diameter of C2C12 myotubes. (**G**-**J**): Protein levels of p-mTOR/mTOR, p-P70S6K/P70S6K, and p-4E-BP1/4E-BP1 in the C2C12 cells. Data are presented as mean ± SE, the * indicates *p* < 0.05

To further investigate whether HME promotes muscle growth through the mTOR signaling pathway, we combined HME with the mTOR inhibitor (Rapamycin) to observe if Rapamycin can partially abolish the muscle-enhancing effects mediated by HME. We found that Rapamycin treatment exhibited a dose-dependent inhibition on C2C12 cell proliferation. Based on these results, we selected 25 nM as the concentration and 24-hour exposure time for the subsequent treatments, ensuring sufficient cell viability and appropriate inhibitory effects (Fig. 5A). Rapamycin was added to differentiated C2C12 myotubes for 24 h. Rapamycin did not exhibit inhibitory effects on well-differentiated C2C12 myotubes compared to the control group. However, Rapamycin significantly suppressed the increase in density, diameter, and length induced by HME in C2C12 myotubes (Fig. 5B-F). Rapamycin also significantly inhibited the phosphorylation of the mTOR-P70S6K-4EBP1 pathway activated by HME (Fig. 5G-J). These findings suggest that the promoting effect of HME on muscle is associated with its ability to activate the mTOR-P70S6K-4EBP1 pathway.

### HME treatment enhances muscle growth and performance associating with related signaling pathways with no effect on liver or kidney function

**Fig. 6:**
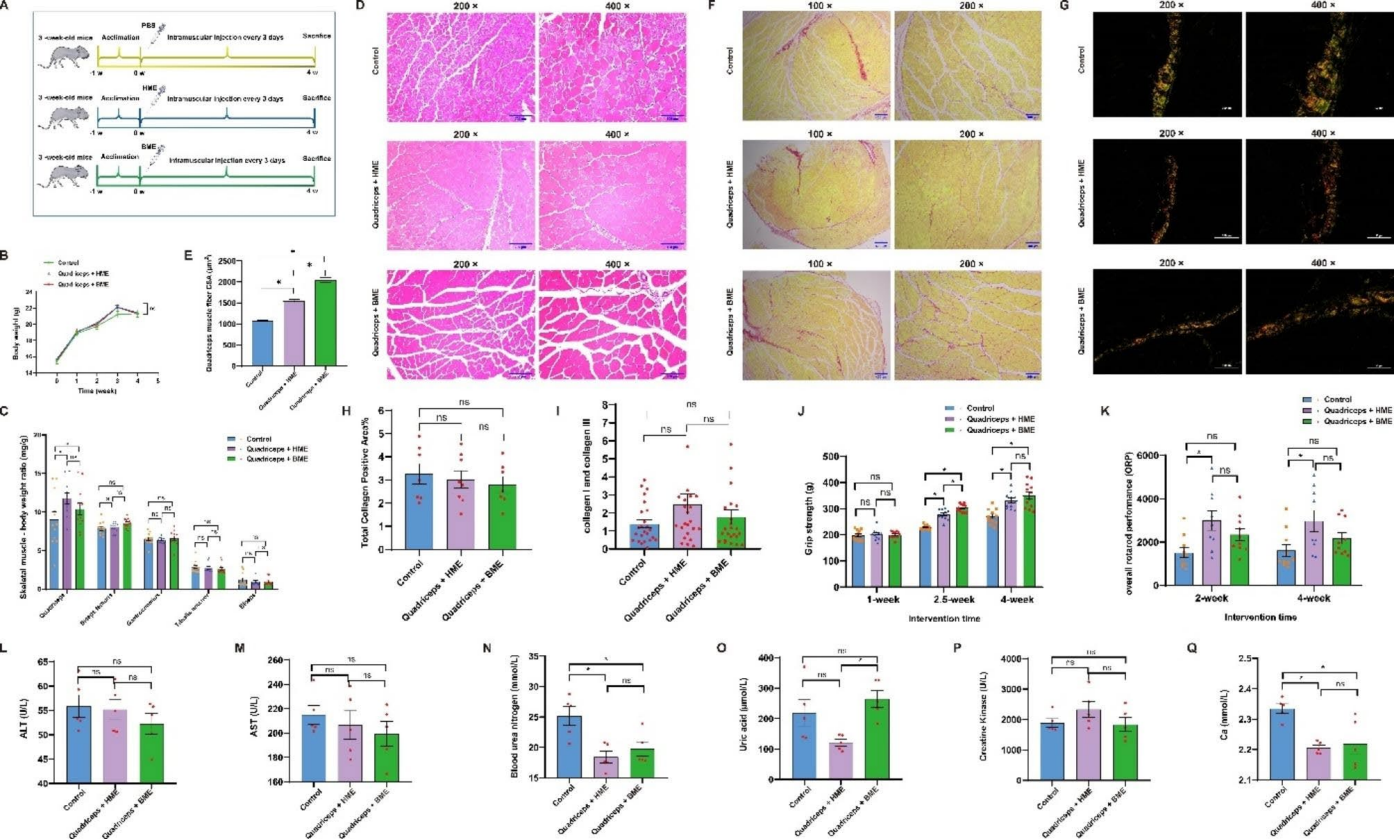
Effects of HME and BME on mouse muscle performance and physiological status. (**A**): Experimental design for animal study. (**B**): Changes in body weight within the experiment. (**C**): Ratio of skeletal muscle mass to body weight. (**D**): H&E staining of cross sections of the quadriceps muscle. (**E**): Cross-sectional area of muscle fibers in the quadriceps muscle. (**F**): Sirius red, without polarized light. (**G**): Sirius red, under polarized light. (**H**): Quantification of the total content of collagen stained by Sirius red. (**I**): The ratio of pixel areas between collagen I and collagen III under polarized light. (**J**): Grip strength test in different groups (n = 6). (**K**): Overall rod performance (ORP) of mice (protocol: 5–45 rpm, 300 s cutoff) (n = 3). (**L**-**Q**): Serum biochemical biomarkers. Data are presented as mean ± SE, the * indicates *p* < 0.05, N = 10–12

Throughout the experiment, weight gain did not differ significantly among the three groups (P > 0.05) (Fig. 6A-B). To investigate the effects of HME and BME on muscle mass in mice after injection, we assessed each group’s skeletal muscle-to-body weight ratio. We observed a significant increase in the weight ratio of the quadriceps in both the HME and BME groups compared to the control group (*p* < 0.05). At the same time, other muscles did not show any significant differences (Fig. 6C). In H&E-stained images. We calculated the average muscle fiber cross-sectional area (CSA) to evaluate muscle fiber size. The HME and BME groups exhibited a significant increase in the average muscle fibers CSA compared to the control group. Further, we examined the morphology of muscle fibers among the three groups through H&E staining. We found that the muscle fibers in the HME group were higher fiber densities than the other two groups. In comparison, those in the BME group were significantly thicker than the other two groups but showed a relatively looser arrangement (Fig. 6D-E). After evaluating with Sirius Red staining, the total collagen area was observed to be similar among different groups. Similarly, no differences were observed in the ratio of collagen I to collagen III between the different groups under polarized light (Fig. 6F-I). To assess the exercise performance, grip strength and rotarod performance were measured in the experimental animals, respectively. The grip strength in the HME and BME groups was significantly higher than that of the control group on the 2.5th and 4th weeks of the experiment (Fig. 6J). After adaptive rotarod training, mice in the HME group exhibited a significant improvement of ORP in the 2nd and 4th weeks, compared to the control group. In contrast, the ORP of mice in the BME group remained unchanged (Fig. 6K).

The evaluation of the experiment’s biochemical analysis serves as a valuable clinical indicator of the health status of the experimental animals, offering insights into their nutrition, protein and muscle metabolism, energy supply, and waste excretion. Neither the HME nor BME interventions significantly altered alanine aminotransferase (ALT) and aspartate aminotransferase (AST), indicators of liver function and injury assessment (Fig. 6L-M). Most amino acids resulting from protein metabolism convert to urea, which the kidneys excrete. Urea nitrogen typically reflects the body’s protein metabolism state. Compared with the control group, serum samples from the HME and BME intervention groups showed a significant decrease in blood urea nitrogen (*p* < 0.05) (Fig. 6N). Creatine kinase and uric acid are generally associated with fatigue. Creatine kinase converts creatine to phosphoprotein, regulating muscle energy supply, while uric acid, primarily produced by the liver and skeletal muscles, is a nucleic acid metabolism product excreted through the kidneys. After HME treatment, there was a downward trend in uric acid and increased creatine kinase, but no significant difference was observed (Fig. 6O-P). Calcium ions combine with myoglobin and myosin during muscle contraction and movement, promoting muscle fiber sliding and contraction. The HME and BME interventions enhance the efficiency of serum calcium utilization, significantly decreasing serum calcium concentration compared with the control group (Fig. 6Q). Overall, HME and BME interventions promote muscle growth, enhance performance, and increase endurance ability to muscle.

**Fig. 7:**
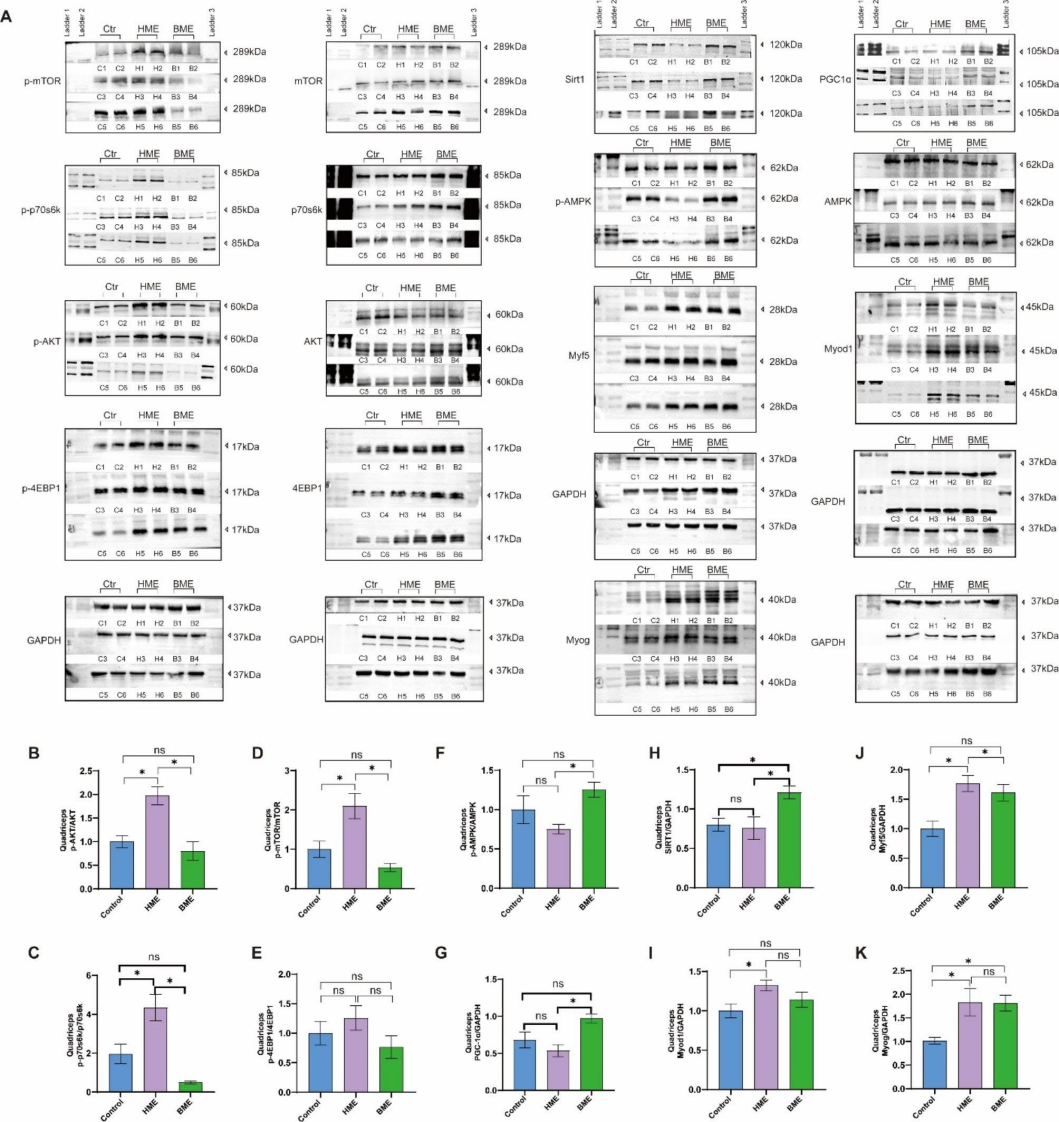
Effects of HME and BME on signaling pathway in mouse quadriceps. (**A**): Figures for Western blotting detection, where C1-C6 lanes for the control group, H1-H6 lanes for the HME group, and B1-B6 lanes for the BME group. (**B**-**E**): Protein levels of p-Akt/Akt, p-mTOR/mTOR, p-P70S6K/P70S6K and p-4E-BP1/4E-BP1 in quadriceps muscle. (**F**-**H**): Protein levels of p-AMPK/AMPK, SIRT1 and PGC-1α in the quadriceps muscle. (**I**-**K**): Protein levels of muscle regulatory factors Myog, Myf5 and Myod1 in the quadriceps muscle. Data are presented as means ± SE, and the * indicates *p* < 0.05

Besides, according to the indication of KEGG analysis, we also investigated and testified this point in muscles of HME and BME groups. Compared to the control group, the HME group exhibited a significant increase in p-AKT/AKT, p-mTOR/mTOR, Myod1, Myf5, and Myog expression levels. However, p-AMPKα/AMPKα, SIRT1, and PGC-1α levels were not affected by HME treatment. On the other hand, BME treatment led to a significant increase in PGC-1α, SIRT1, and Myog levels compared to the control group, with no significant effects observed in p-AKT/AKT and p-mTOR/mTOR levels (Fig. 7). These data also showed similar fashion with C2C12 cells after the intervention.

### Effect of HME and BME intervention on the amino acid profile of quadriceps

#### Sample quality control and data analysis

The comprehensive analysis of UHPLC-MS/MS exhibits high sensitivity and specificity in detecting various amino acids and their metabolites, ensuring accurate material quantity determinations. The UHPLC system effectively separates and elutes diverse amino acids and their metabolites from the quadriceps of all three groups, achieving results within a short span of 12 min (**Fig. S7A**). The MWDB database (Metware Database, Metware Bio-technology Ltd, Wuhan, China), which was also constructed using previously-established samples, facilitated the qualitative analysis of mass spectrometry data. We also used the Multiple Reaction Monitoring (MRM) mode of triple quadrupole mass spectrometry for quantification. Following the acquisition of mass spectrometry analysis data from diverse samples, we could integrate chromatographic peaks of all target compounds, perform quantitative analysis using the standard curve method, and ensure quality control through multiple QCs. The results of overlapping total ion chromatograms (TIC) from varied sample analyses and high Pearson correlation coefficients (R2) between QC samples, with proportions of QCs having a coefficient of variation (CV) less than 0.2 exceeding 80%, indicate exceptionally stable experimental data (**Fig. S7A-C**).

#### Profiling of amino acid and metabolites in quadriceps from control, BME, and HME groups through clustering and visualization analyzing methods

**Fig. 8:**
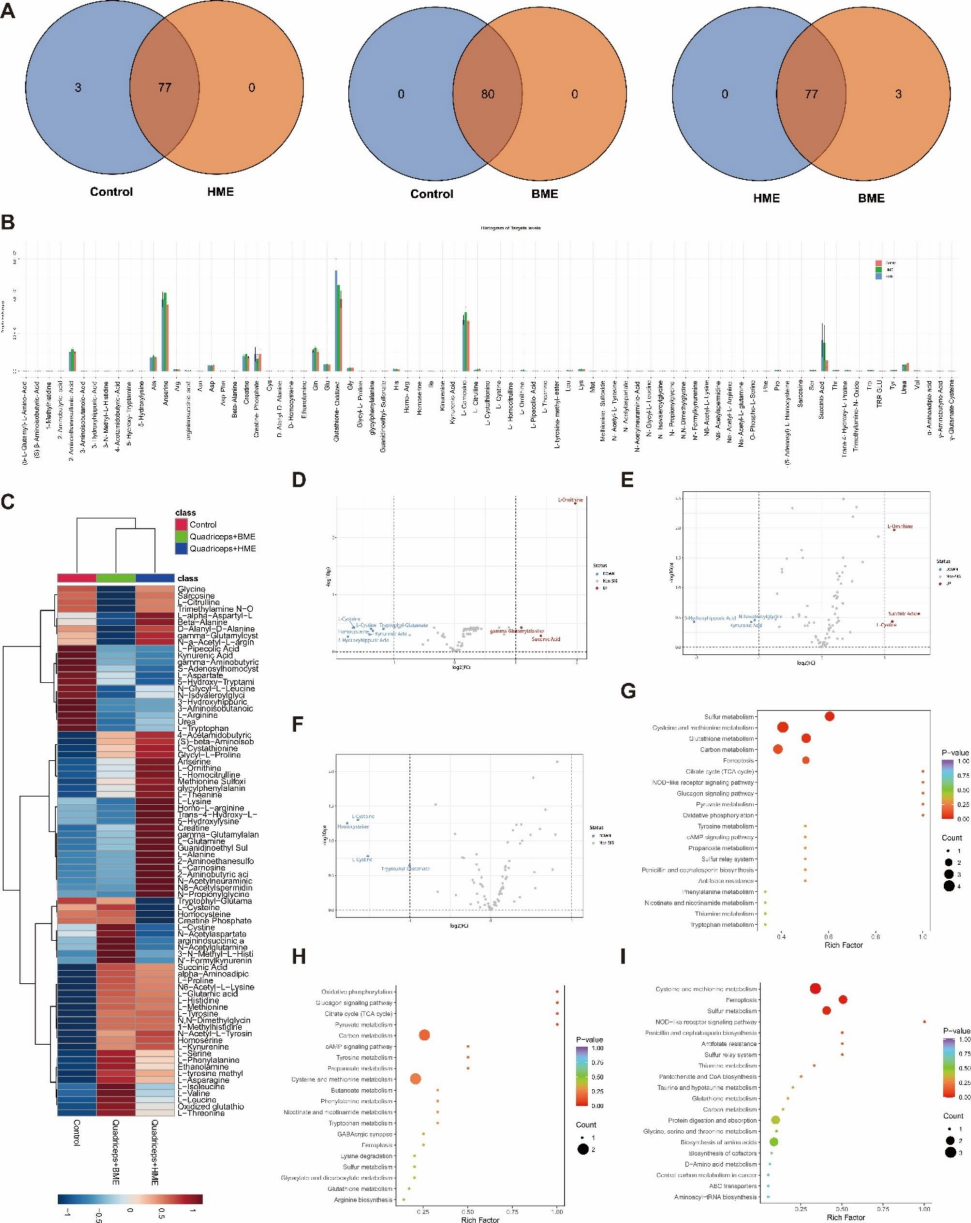
The pictures for Amino acid profiling in mouse quadriceps by targeted metabolomics analysis. (**D**): Venn diagram of amino acid composition in quadriceps of three groups of mice. (**I**): Bar chart representing the content of amino acids and their metabolites in the quadriceps muscle of three groups of mice. (**J**): Heat map illustrated the relative abundance distribution of amino acids and their metabolites in the quadriceps muscle of three groups of mice. (**K**): Volcano plot analysis of differentially abundant metabolites between the HME and control groups. (**L**): Volcano plot analysis of differentially abundant metabolites between the BME and control groups. (**M**): Volcano plot analysis of differentially abundant metabolites between the HME and BME groups. (**U**): KEGG enrichment analysis of differentially abundant metabolites between the HME and control groups. (**V**): KEGG enrichment analysis of differentially abundant metabolites between the BME and control groups. (**W**): KEGG enrichment analysis of differentially abundant metabolites between the HME and BME groups

We detected 80 types of amino acid and metabolite species in the quadriceps samples of the control and BME groups, while the HME group showed 77 species, which were already identified in either control or BME groups (Fig. 8A). The PCA analysis revealed distinct separation among the amino acid components of the three groups (**Fig. S8A**). Further investigation was performed using the OPLS-DA model, a supervised discriminant analysis method. The OPLS-DA score plot displayed complete separation between the control and HME groups, control and BME groups, and HME and BME groups, underscoring significant differences in amino acids and their metabolites among these groups (**Fig. S8B-D**). In order to illustrate the differences in substance contents among the three groups, we generated bar graphs and a table displaying amino acid and metabolite contents (Fig. 8B **and Table S4**). Heat maps were also created to display the relative distribution of average values of amino acids and their metabolites among the three groups. Hierarchical clustering grouped the HME and BME groups together, suggesting that the pattern of distribution of amino acids and their metabolites was similar to each other and different from the control group (Fig. 8C).

#### Differential metabolites screening using volcano plot, z-score analysis and random forest models analysis

Using the volcano plot analysis, metabolites that demonstrated significant intergroup differences with fold change (FC) greater than 2 (up-regulated) or FC less than 0.5 (down-regulated) compared to the control group were identified as important features. In the quadriceps from the HME group, the levels of L-Ornithine, Succinic Acid, and γ-Glutamate-Cysteine were significantly increased compared to the control group. Similarly, in the BME group, significant elevations in the levels of L-Ornithine, Succinic Acid, and L-Cystine were observed in the quadriceps when compared to the control group. L-Ornithine aids in reducing the buildup of toxic metabolites produced by amino acid metabolism in muscle tissue by engaging in the urea cycle, thereby facilitating urea metabolism and excretion and to some extent, preventing muscle tissue damage and fatigue. Moreover, L-Ornithine enhances the production of dopamine and γ-amino butyric acid (GABA) metabolites, thereby augmenting physical performance. The former primarily aids in muscle contraction and motor control processes, reinforces muscle strength and endurance, and boosts fat metabolism and burning, while the latter mainly engages in neural transmission and inhibition processes, reduces tension and stress, and facilitates muscle relaxation and recovery. Succinic acid serves as an intermediary in the tricarboxylic acid (TCA) cycle, a prominent intermediate source, and an energy supply for amino acid synthesis and transport, in addition to participating in the energy metabolism process inside muscle cells. γ-Glutamate-Cysteine is a small molecule compound comprised of sulfur elements that function as an antioxidant in skeletal muscles by participating in the glutathione antioxidant pathway, thus protecting skeletal muscle tissue from oxidative damage. L-Cystine, a sulfur-containing amino acid and a constituent of protein structures, necessitates optimal levels to support muscle growth and repair (Fig. 8D-F).

To better understand the amino acid and metabolite profiles present in the quadriceps from HME and BME groups, a random forest model was constructed. The results demonstrated that L-Ornithine and Urea were critical in distinguishing the HME and BME groups from the control group. Hence, L-Ornithine’s involvement in the urea cycle and its ability to promote urea metabolism and excretion make it a pivotal molecular target for HME or BME to influence mouse skeletal muscle function. Furthermore, L-Lysine also emerged as the most significant variable between the HME and BME groups. L-Lysine takes part in diverse processes relevant to skeletal muscles as an essential amino acid. It can boost muscle growth and ensure skeletal muscle health and function maintenance. In addition to this, L-Lysine also can facilitate calcium absorption and utilization, thereby augmenting skeletal muscle strength and vitality (**Fig. S9A-D**).

#### KEGG annotation and enrichment analysis

Metabolite data demonstrating significant differences between groups were annotated and categorized using KEGG. The KEGG metabolic pathway (ko01100) was found to be the most frequently annotated (**Fig. S10A-C**). KEGG enrichment analysis was performed on the different metabolites and overall changes in metabolic pathways were analyzed. In both the HME group and control group, there was significant enrichment of the different metabolites in the TCA cycle pathway, an essential pathway for energy metabolism and biomolecule synthesis in the human body. As muscle movement requires substantial ATP support, the AKT-mTOR-p70s6k-4EBP1 pathway is closely connected to the TCA cycle since it facilitates anabolic processes that necessitate energy derived from this cycle. The BME and control groups showed significant enrichment of the difference metabolites in the oxidative phosphorylation pathway, one of the most critical energy production mechanisms in mitochondrial in the human body, providing an ample supply of ATP to maintain muscle contraction and function. This pathway also regulates mechanisms responsible for maintaining skeletal cell metabolic state and healthy function, such as mitochondrial membrane potential and antioxidant defense. The AMPK-SIRT1-PGC-1α pathway is also closely linked to the oxidative phosphorylation pathway, as it promotes mitochondrial biogenesis and oxidative metabolism. The HME and BME groups demonstrated significant enrichment of the different metabolites in the NOD-like receptor signaling pathway, a significant immune regulatory pathway. NLRs are important receptor proteins active within the cytoplasm of cells, able to sense various stimuli from bacteria, viruses, and cell stress. It maintains body homeostasis by regulating inflammatory reactions, apoptosis, autophagy, and other reactions. The NLR signaling pathway also participates in skeletal muscle growth, repair and metabolism, with NLRP3 as a common NLR significantly impacting skeletal muscle’s health and function. Over-activation may lead to phenomena, including inflammatory reactions, oxidative stress, mitochondrial dysfunction, and muscle atrophy (Fig. 8G-I).

### Association analysis between the amino acid profiles in HME and BME and amino acid profiles of quadriceps after intervention

**Fig. 9:**
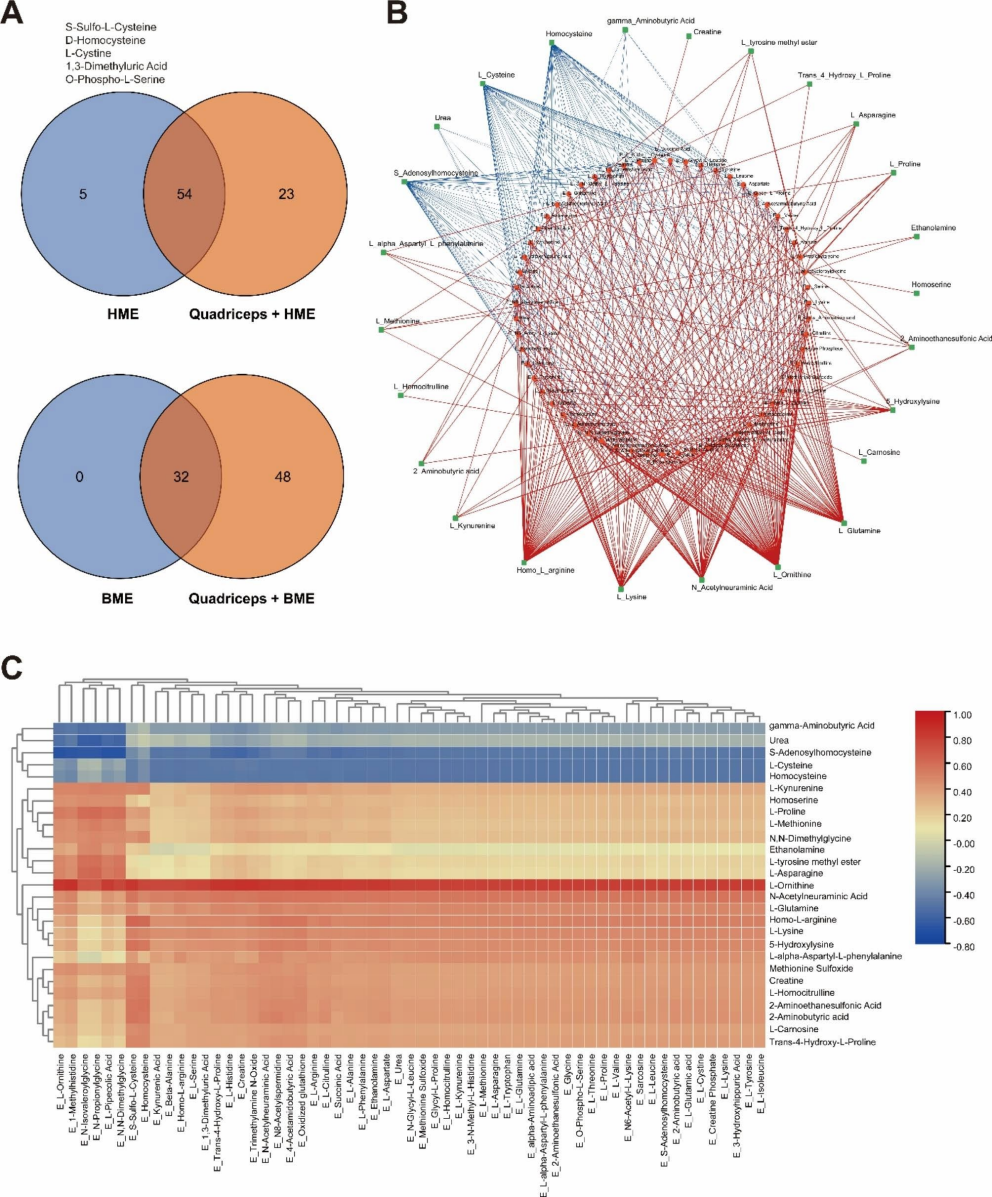
Correlation analysis between the contents of HME and BME and the amino acid profile of the intervened mouse quadriceps. (**A**): Venn diagram showing the amino acid composition of quadriceps and corresponding HME and BME. The five amino acids (S-Sulfo-L-Cysteine, D-Homocysteine, L-Cystine, 1,3-Dimethyluric acid, and O-Phospho-L-Serine) are in HME but not in quadriceps muscle after HME intervention that is listed in the upper left corner. (**B**): Correlation network picture of milk EVs and amino acid composition of the intervened quadriceps muscle in mice. The red lines indicate significant positive correlations. The blue lines indicate significant negative correlations. The thicker the line indicates, the stronger the correlation. (**C**) Heat map of the correlation between milk EVs and amino acid composition of intervened quadriceps muscle in mice

To identify the critical amino acids in HME and BME that contribute to skeletal muscle growth, and performance in mice, we conducted an association analysis between these EVs compounds and amino acid profiles in the quadriceps after treatment. After Venn analysis, we noted that five amino acids and their metabolites in HME were not included in the quadriceps muscles of mice intervened with HME, while all amino acids and their metabolites in BME were detected in the quadriceps muscles of mice intervened with BME (Fig. 9A). Pearson correlation coefficients demonstrated significant correlations (*p* < 0.05) between certain amino acids of EVs and the amino acid profiles of the quadriceps following HME and BME interventions. By combining these findings, we constructed a correlation network and heat map (Fig. 9B-C), which revealed that L-Ornithine of the EVs exhibited the strongest positive correlation with amino acid contents in the quadriceps, while several other amino acids, including Homo-L-arginine, L-Glutamine, N-Acetylneuraminic Acid, and L-Lysine of EVs, also displayed strong positive correlations with amino acid contents in the quadriceps muscles. Homo-L-arginine, an amino acid derivative similar to arginine, promotes muscle contraction and enhances muscle strength. L-Glutamine, on the other hand, is able to convert into glutamic acid within muscle cells and contributes to the synthesis of proteins that aid in muscle growth and repair. Meanwhile, N-Acetylneuraminic Acid plays a crucial role in assisting the synapses between neurons and skeletal muscles and also contributes to maintaining neuromuscular connections, ultimately promoting muscle contraction. L-Lysine is another significant substrate for muscle protein synthesis. Thus, it is also involved in promoting muscle growth and repair. These amino acids may be integral in determining the molecular targets for HME or BME’s effect on mouse skeletal muscle function. Furthermore, several amino acids and their metabolites, such as S-Sulfo-L-Cysteine, D-Homocysteine, L-Cystine, γ-Aminobutyric Acid, and Urea, displayed significant negative correlations with certain amino acid contents of HME and BME. Interestingly, they partially overlapped with the five amino acids and their metabolites that were not included in the quadriceps muscles of mice after the HME intervention. These findings may relate to the clearance of toxic amino acid metabolites in skeletal muscles or the increased consumption of amino acids necessary for muscle growth and metabolism.

### Investigation of the correlation between quadriceps amino acid profile and biochemical markers/muscle metabolic signaling pathways in mice using Pearson correlation analysis

**Fig. 10:**
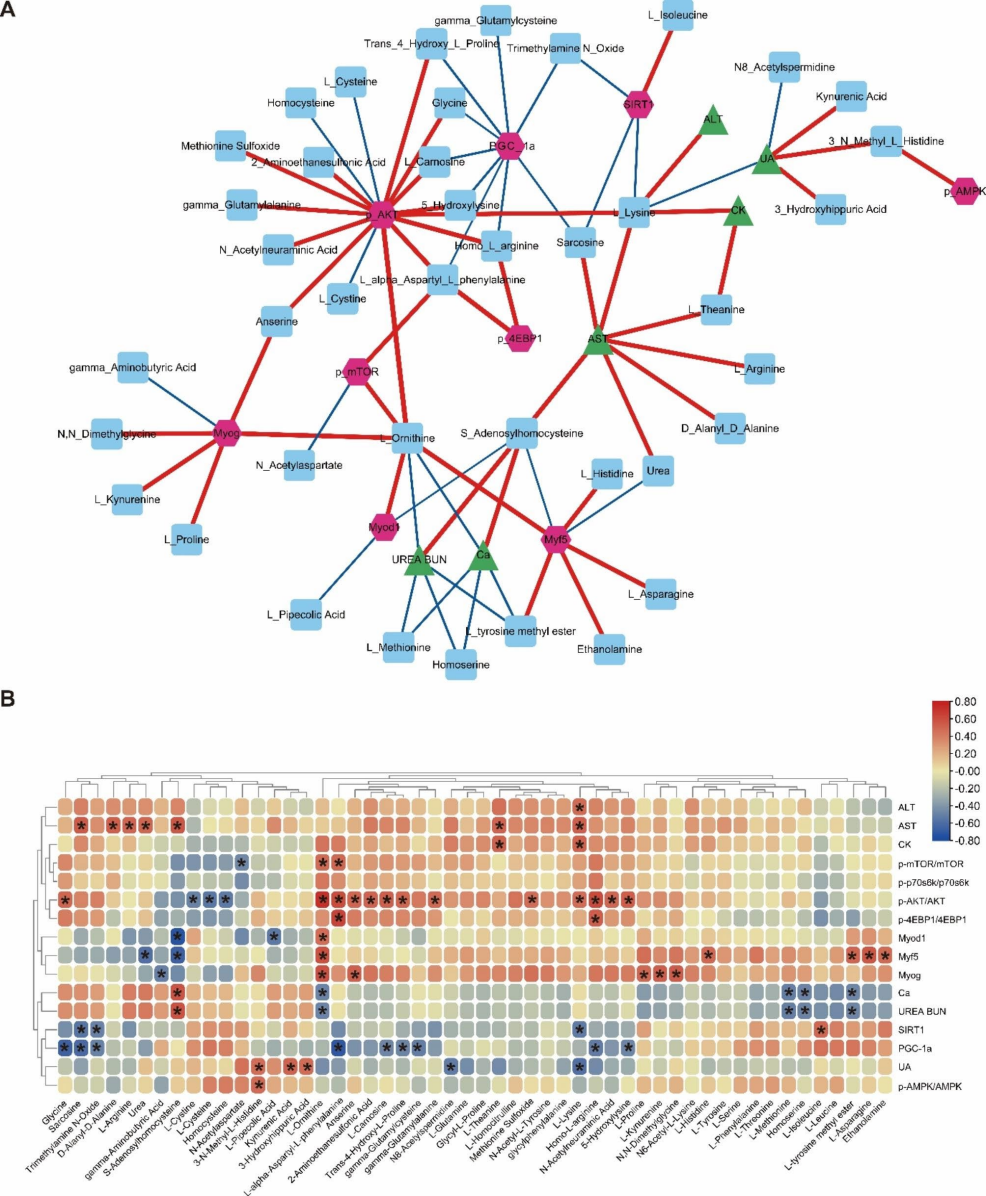
Correlation analysis of amino acid profiling in mouse quadriceps with biochemical markers/muscle metabolic signaling. (**A**): Network diagram showed the correlation between amino acid profiling in mouse quadriceps and biochemical markers/muscle metabolic signaling. The red lines represented a significant positive correlation, and the blue lines represented a significant negative correlation. The thicker the line indicated, the stronger the correlation. (**B**): Heat map showed the correlation between amino acid profiling in mouse quadriceps muscle and biochemical markers/muscle metabolic signaling. The * indicates *p* < 0.05

We screened and constructed a correlation network and heat map of significant indicators to investigate the relationship between amino acid distribution patterns and biochemical markers/muscle metabolic signals in mice. Notably, L-Ornithine displayed the strongest correlation with biochemical markers/muscle metabolic signals in mice, exhibiting a significant positive association with the activation of the AKT/mTOR pathway and MRF. Moreover, the p-AKT demonstrates the most significant positive correlation with quadriceps amino acids (Fig. 10). These findings suggest that the distribution pattern of certain amino acids in mouse muscles can influence the expression levels of biochemical markers and muscle metabolic signals.

## Discussion

Skeletal muscle, the largest visceral organ in mammals, plays a critical role in supporting various movements and regulating whole-body metabolic homeostasis [45]. Peak human skeletal muscle mass is typically achieved during young adulthood, typically in the third decade of life. Subsequently, a gradual decline in muscle mass with aging leads to reduced strength and mobility in older individuals. Beginning in middle age, around 50% of muscle fibers are lost from limb muscles, starting at around 50 years old and continuing until 80 years old [46–48]. The DoHad hypothesis indicated that breeding styles, body/nutrition status or stress conditions of early life are closely relative to adult health and disease [49]. Therefore, enhancing childhood skeletal muscle growth and development is essential for normal body functioning, including movement, posture, and metabolic regulation, as it establishes a foundation for sustained health and physical ability. Nutrition is a crucial factor in muscle development during infancy, and breast milk has been identified as the most important functional activator to promote infant body growth. However, consuming bovine milk and its dairy products over extended periods can contribute to civilization’s diseases and negatively impact human health. These products activate mTORC1 and milk-derived growth factors, stimulating growth and proliferation and creating a complex signaling network throughout the body [50]. While increased mTORC1 activity can negatively impact various tissues and promote aging, it is essential for preventing age-related muscle loss [51].

A marked discrepancy exists in milk’s growth-promoting effect across various animal species. For instance, an infant intakes 180 days of human milk to double increase their weight, while calves can achieve this feat in as little as 40 days. Humans and other primates enjoy the evolutionary advantage of slow growth, which confers specific benefits for complex brain development. Nevertheless, modern-day humans have negated this evolutionary edge through unregulated formula feeding and persistent milk consumption [52]. As such, selecting nutritional intervention modes alongside targeted, meticulous delivery is especially critical for human health. Mobley et al. have demonstrated that supplementing C2C12 myotubes cultures with EVs derived from bovine milk enhances myotubes growth, potentially indicating a connection between milk intake and augmented muscle protein [11]. Nonetheless, conflicting results from other studies may be influenced by factors like low distribution within skeletal muscles prompted by oral EVs administration [12, 13]. Administration routes, including intravenous (iv), oral (po), subcutaneous (sc), intranasal (in), and intramuscular (im), have substantial impacts on biological distribution [53], with intramuscular injection displaying robust and enduring accumulation at the injection site while minimizing extensive systemic dispersion [54]. Consequently, we utilized intramuscular injection into the quadriceps muscle for precise delivery of milk EVs in our study. Moreover, various types of collagen, primarily Type I and Type III collagen, are typically present in skeletal muscles, playing an important role for maintaining muscle morphology and function. However, excessive deposition of collagen proteins can lead to tissue fibrosis, impairing grip strength, while a substantial increase in Type I collagen content can significantly elevate tissue stiffness, restricting skeletal muscle extension and contraction [55, 56]. Nevertheless, our research findings indicate that interventions with HME and BME have no impact on the content of collagen proteins within skeletal muscles or the distribution of collagen types, thereby posing no risk of tissue fibrosis. We provide evidence that both HME and BME can boost myofibers’ growth and heighten mice’s quadriceps weight ratio and grip strength. Of the two EVs types, HME stands out as it consistently bolsters myotubes growth by continuously instigating mTOR pathway activation inside muscles. Meanwhile, BME’s influence on myotubes synthesis metabolism may arise from the transient activation of early-stage mTORC1 signaling activation [11]. Furthermore, HME intervention significantly enlarges C2C12 myotubes’ diameter and length, presenting a more comprehensive differentiation state, whereas BME only increases myotubes’ diameter. While myofibers in the quadriceps of the BME group are thicker than those in the HME group, the former’s arrangement is looser compared to the latter’s tighter disposition. These observations mirror humans’ gradual growth phenomenon and calves’ rapid growth. Moreover, given HME’s propensity for sustained mTORC1 activation in skeletal muscles, its potential for averting age-induced muscle loss is substantial.

Amino acids represent the fundamental building blocks of proteins and are critical for muscle growth and repair. Intramuscular injection of amino acids provides direct entry into the bloodstream, thereby supplying various tissues and organs throughout the body via circulation [57]. Although this method is advantageous in providing rapid amino acid supplementation, excessive amino acid injection can lead to potential side effects such as gastrointestinal discomfort, cardiovascular system reactions, and renal impairment [58–60]. Additionally, single amino acid administration may produce undesirable outcomes, including competitive absorption, toxicity, and resistance. Conversely, multiple amino acids working synergistically can maximize their beneficial effects [61]. Our research has identified abundant amino acids in milk EVs, particularly in HME. The HME sample contained 59 types of amino acids and metabolites, whereas the BME sample had 32 types of amino acids and metabolites, with HME encompassing all amino acid categories present in BME. Milk EVs are Nano-sized particles containing multiple bioactive molecules originating from mammary epithelial cells, macrophages, lymphocytes, and even cells from other body parts, circulating through the blood to breast milk [62]. Essentially, milk EVs possess many attributes of drug delivery vehicles, including good host tolerance, longer half-life, internalization by other cells, carrying large amounts of endogenous cargo, low invasiveness, no immunogenicity, high safety and efficacy, and low cost [14]. After intramuscular injection, EVs deposit within cells surrounding the injection site and interact with surrounding cells to exert their effects. Additionally, numerous studies indicate that intramuscular injection of EVs leads to significant signal accumulation at the injection site for a period of up to three days or even longer [54]. As such, HME, enriched with multiple amino acids, represents an ideal nutritional supplement for promoting skeletal muscle growth and repair. Additionally, both HME and BME interventions induced significant changes in the amino acid spectra of the quadriceps muscles in mice compared to the control group.

To investigate the impact and correlation of HME or BME contents on mouse skeletal muscle growth and performance, we conducted a correlation analysis of the amino acid spectra in the quadriceps muscles of mice with HME or BME. Among them, L-Ornithine of quadriceps was positively correlated with the amino acid content of HME and BME and showed the strongest correlation with biochemical markers and muscle metabolic signals in mice. Furthermore, significant positive correlations were found between L-Ornithine and the activation of the AKT/mTOR pathway and MRF, suggesting that L-Ornithine may be a critical molecular target for the effect of HME or BME on mouse skeletal muscle growth and performance. L-Ornithine is a metabolite of L-Arginine, one of the most important components of urea amino acids in the urea cycle. Nearly all therapeutic applications of L-Arginine also apply to L-Ornithine because it can more easily enter mitochondria to effect cells [63], and supplementation with L-Ornithine is easier to absorb than L-Arginine [64]. In addition, previous studies have shown that the amino acids in milk EVs are derived from blood. Interestingly, the level of ornithine in human breast milk is significantly higher than that in the milk of other mammals [65], and infant formula does not contain ornithine [66]. Our research findings are consistent with previous studies showing significant ornithine absorption by mammary glands in cows, goats, sows, rabbits and horses, resulting in lower or no ornithine in milk [67–70]. Our results also demonstrated significantly higher levels of L-Ornithine and L-Arginine in HME than in BME. After interventions with HME or BME, the level of L-Arginine in the quadriceps muscle decreased, while L-Ornithine significantly increased compared to the control group. Additionally, the level of L-Ornithine in the quadriceps muscle was significantly higher in the HME group than in the BME group. These results suggest that the level of L-Ornithine in mouse skeletal muscles is not solely derived from HME or BME but may be influenced by their ability to promote the conversion of L-Arginine to L-Ornithine in skeletal muscles.

Urea cycle-related amino acids are unique in their inclusion of urea nitrogen, facilitating the conversion and elimination of waste products resulting from amino acid metabolism and regulation of nitric oxide levels in blood [70]. Within skeletal muscles, these properties promote L-Ornithine’s ability to support muscle function by reducing toxic metabolic byproducts, such as ammonia, that accumulate in muscle cells during intense exercise [71–73]. The resulting increase in nitric oxide can enhance blood flow to working muscles during exercise and promote the production of metabolites like dopamine and GABA, improving muscle function and reducing fatigue [74, 75]. Additionally, L-Ornithine is vital in supporting muscle growth and repair, reducing fat, improving muscle quality, and enhancing exercise capacity in skeletal muscles [64]. The combination of L-Ornithine and L-Arginine stimulates growth hormone release, supporting growth in children with congenital or nutritional deficiencies and suppressing sarcopenia development in older adults [64, 76]. Furthermore, L-Ornithine plays a critical role in creatine metabolism, a key molecule for muscle energy production, thereby contributing to improved muscle quality and exercise capacity [77]. Studies also demonstrate ornithine’s ability to increase lean body mass, grip strength, overall rod performance, and other physical attributes in mice [78–80]. These findings further confirm our results that HME contains a rich source of amino acids, particularly L-Ornithine, which significantly enhances growth and exercise capacity in mouse skeletal muscles. Milk EVs, which serve as natural carriers of endogenous biomolecules, offer distinct advantages over other drug delivery vehicles. Consequently, HME also has considerable potential as a promising carrier for treating musculoskeletal diseases, warranting further investigation. Given its outstanding protective effect on skeletal muscles, HME can serve not only as a supplement to support the growth and development of infants and young children, but also as a means to protect muscle mass in adults, particularly among older adults and athletes. Moreover, given that L-Ornithine and L-Arginine are conditionally essential amino acids, their endogenous synthesis may only partially meet daily requirements. Therefore, an inadequate endogenous supply of these amino acids may limit maximal growth in rapidly growing infants. Previous studies have demonstrated that a lack of L-Arginine in pig’s milk can restrict the maximum growth rate of neonatal pigs, while a deficiency of L-Arginine in food can delay puberty and maturity in rats [64, 69, 70, 81]. Related factors, including excessive ammonia production, rapid growth, pregnancy, trauma, protein deficiency, or malnutrition, may result in a shortage of urea amino acids. Otherwise, L-Ornithine, a metabolite of arginine, is more easily absorbed than arginine with higher utilization efficiency. A regular diet typically provides about 5 g of L-Ornithine daily to fulfill the body’s requirements, with higher doses necessary for therapeutic purposes [64, 82, 83]. Furthermore, milk from other mammals contains significantly lower levels of L-Ornithine than human milk, and L-Ornithine is not present in formula milk. Our findings thus suggest that adding ornithine to infant formula or using HME supplements may be an effective strategy to enhance growth in non-exclusively breastfed infants. Although L-Ornithine has been identified as a potential molecular target through preliminary bioinformatics analysis in this study, future research should primarily focus on experimental validation of the effects of L-Ornithine and other identified targets to gain a comprehensive understanding of their contributions to the impact of HME or BME on skeletal muscle.

## Conclusion

Our study revealed that milk-derived EVs contain various amino acids, but the distribution of these amino acids differs significantly between HME and BME. Notably, HME showed higher levels of Creatine-phosphate and L-Ornithine compared to BME. Local injection of HME into the quadriceps promoted muscle growth and improved overall rod performance in mice, with persistent activation of the AKT-mTOR-p70s6k signaling pathway and expression of myogenic factors observed specifically for HME. We suggest that L-Ornithine present in HME may serve as a crucial indicator influencing skeletal muscle function in mice. This is the first study to investigate milk EVs containing bioactive constituents capable of stimulating skeletal muscle growth, performance, or anabolic metabolism, which provides novel insights and reconciles conflicting outcomes from previous studies. Our findings also provide the first direct or indirect evidence of the regulatory role of amino acid contents within HME on skeletal muscle growth and performance.

### Electronic supplementary material

Below is the link to the electronic supplementary material.

Supplementary Material 1

Supplementary Material 2

Supplementary Material 3

## Data Availability

Data will be made available on request.

## Abbreviations

HME: Human milk extracellular vesicles
BME: Bovine milk extracellular vesicles
NTA: Nanoparticle tracking analysis
TEM: Transmission electron microscopy
ALT: Alanine aminotransferase
AST: Aspartate aminotransferase
QC: Quality control
TIC: Total ion chromatograms
PCA: Principal component analysis
OPLS-DA: Orthogonal partial least squares discriminant analysis
CSA: Cross-sectional area
