# Supplementary material for: Human milk extracellular vesicles enhance muscle growth and physical performance of immature mice associating with Akt/mTOR/p70s6k signaling pathway
Source: J Nanobiotechnology. 2023 Aug 29;21:304. doi: 10.1186/s12951-023-02043-6 (PMC10463453; doi:10.1186/s12951-023-02043-6)
Supplement: Supplementary file 1 — Supplementary Material 1 [file 12951_2023_2043_MOESM1_ESM.docx]

**Supplemental Table**

**Table S1. Information of human milk donors**

| **Milk Donors number** | **Age (Years)** | **Number of Previous Deliveries** | **Gestational age (weeks)** | **Birth weight (g)** | **Milk (mL)** | **Infant age at milk collection (d)** |
| --- | --- | --- | --- | --- | --- | --- |
| 1 | 28 | 1 | 41+0 | 3190 | 200 | 42 |
| 2 | 28 | 2 | 39+6 | 2710 | 200 | 42 |
| 3 | 28 | 1 | 39+0 | 3750 | 200 | 42 |
| 4 | 30 | 1 | 39+5 | 2970 | 200 | 42 |
| 5 | 32 | 2 | 39+0 | 3100 | 200 | 42 |
| 6 | 30 | 1 | 39+1 | 3570 | 200 | 42 |
| 7 | 32 | 1 | 38+0 | 3390 | 200 | 42 |
| 8 | 30 | 1 | 40+0 | 3150 | 200 | 42 |
| 9 | 29 | 2 | 39+2 | 3130 | 200 | 42 |
| 10 | 28 | 1 | 38+4 | 3720 | 200 | 42 |

**Table S2. Information of size /concentration for HME and BME**

| **EVs category** | **Mean (nm)** | **Mode (nm)** | **SD (nm)** | **D10 (nm)** | **D50 (nm)** | **D90 (nm)** | **Concentration (particles/ml)** |
| --- | --- | --- | --- | --- | --- | --- | --- |
| HME | 179.2 ± 1.5 | 99.5 ± 1.1 | 72.1 ± 1.2 | 97.0 ± 1.0 | 169.2 ± 2.1 | 284.2 ± 3.4 | 5.18e+11 ± 9.67e+08 |
| BME | 118.6 ± 0.4 | 65.6 ± 1.5 | 59.0 ± 1.0 | 61.5 ± 1.0 | 101.1 ± 1.6 | 195.3 ± 5.7 | 3.91e+11 ± 1.39e+10 |

**Note:** The size /concentration for HME and BME are expressed as mean ± standard error.

**Table S3. Different concentrations of amino acids in HME and BME**

| **Amino acids**  **(compounds: full name)** | **Amino acids**  **(Index: abbreviation)** | **Concentrations in HME (ng/mg)** | **Concentrations in BME (ng/mg)** | ***P* values**  **(HME vs BME)** | **RT** | **Standard curves** | **r** |
| --- | --- | --- | --- | --- | --- | --- | --- |
|  |  |  |  |  |  |  |  |
| 1,3-Dimethyluric Acid | 1,3_Dimethyluric_Acid | 1089.30±118.60 | 0.00 | 7.83E-04 | 1.73 | y = 5.22169e-4 x | 0.99386 |
| 1-Methylhistidine | 1_Methylhistidine | 193.66±4.39 | 103.26±0.46 | 4.09E-05 | 9.32 | y = 0.02095 x - 1.66538 | 0.99258 |
| 2-Aminobutyric acid | 2_Aminobutyric_acid | 166.74±10.83 | 0.00 | 1.09E-04 | 7.51 | y = 0.00365 x - 0.07727 | 0.99827 |
| 2-Aminoethanesulfonic Acid | 2_Aminoethanesulfonic_Acid | 10204.00±498.33 | 46.02±0.55 | 4.15E-05 | 6.99 | y = 0.00541 x - 0.13652 | 0.99783 |
| 3-Hydroxyhippuric Acid | 3_Hydroxyhippuric_Acid | 145.47±5.43 | 4.43±0.28 | 1.68E-05 | 2.36 | y = 0.00325 x | 0.99717 |
| 3-N-Methyl-L-Histidine | 3_N_Methyl_L_Histidine | 1008.20±9.27 | 0.00 | 9.37E-14 | 10.16 | y = 0.00318 x - 0.14133 | 0.99895 |
| 4-Acetamidobutyric Acid | 4_Acetamidobutyric_Acid | 38.35±4.99 | 0.00 | 1.54E-03 | 1.26 | y = 0.00410 x - 0.01513 | 0.99795 |
| L-Alanine | Ala | 8698.50±461.72 | 648.25±12.13 | 7.04E-05 | 8.13 | y = 7.00977e-4 x + 0.03767 | 0.99004 |
| L-Arginine | Arg | 14058.00±345.85 | 1360.90±78.50 | 2.61E-06 | 10.25 | y = 0.00628 x - 0.34310 | 0.99678 |
| L-Asparagine Anhydrous | Asn | 1165.90±35.51 | 0.00 | 4.79E-06 | 9.09 | y = 6.93353e-4 x - 0.02473 | 0.99877 |
| L-Aspartate | Asp | 7984.10±372.16 | 519.88±45.61 | 4.49E-05 | 9.87 | y = 3.41914e-4 x + 0.01017 | 0.99895 |
| L-α-Aspartyl-L-phenylalanine | Asp_Phe | 63.24±3.01 | 0.00 | 3.76E-05 | 8.67 | y = 0.00141 x - 0.02174 | 0.9995 |
| Beta-Alanine | Beta_Alanine | 278.37±34.70 | 0.00 | 1.31E-03 | 8.12 | y = 4.79772e-5 x - 2.37806e-4 | 0.99855 |
| Creatine | Creatine | 6595.20±351.29 | 1675.10±20.59 | 1.56E-04 | 8.14 | y = 0.00904 x - 0.27868 | 0.99822 |
| Creatine Phosphate | Creatine_Phosphate | 2664038.00±130479.00 | 73168.00±35712.00 | 5.12E-05 | 10.89 | y = 7.66350e-7 x + 0.00564 | 0.99163 |
| D-Homocysteine | D_Homocysteine | 29.36±14.84 | 0.00 | 5.45E-04 | 6.69 | y = 5.98736e-4 x - 0.01455 | 0.99962 |
| Ethanolamine | Ethanolamine | 1395.70±98.14 | 91.64±4.37 | 1.90E-04 | 5.30 | y = 8.02998e-4 x + 0.03754 | 0.99704 |
| L-Glutamine | Gln | 1828.10±92.80 | 0.00 | 4.66E-05 | 8.94 | y = 5.15643e-4 x - 0.02262 | 0.99431 |
| L-Glutamic acid | Glu | 387244.00±25318.00 | 1318.80±9.08 | 1.13E-04 | 9.34 | y = 3.85226e-4 x + 0.01614 | 0.99463 |
| Glutathione Oxidized | Glutathione_Oxidized | 55518.00±6266.10 | 1721.20±1605.90 | 1.14E-03 | N/A | y = 2.92472e-6 x + 0.02129 | 0.99101 |
| Glycine | Gly | 2561.40±127.93 | 0.00 | 4.41E-05 | 8.59 | y = 4.21547e-5 x + 0.00231 | 0.99833 |
| Glycyl-L-Proline | Glycyl_L_Proline | 33.95±0.40 | 0.00 | 2.76E-13 | 9.03 | y = 0.01487 x - 0.36031 | 0.99854 |
| L-Histidine | His | 7858.10±204.18 | 1433.30±17.98 | 6.41E-06 | 10.06 | y = 0.00147 x - 0.09597 | 0.99791 |
| Homo-L-arginine | Homo_Arg | 274.65±6.84 | 0.00 | 9.84E-07 | 10.10 | y = 0.00831 x - 0.38932 | 0.99875 |
| L-Isoleucine | Ile | 1123.80±61.89 | 33.30±0.33 | 6.78E-05 | 5.30 | y = 0.00836 x - 0.18023 | 0.99882 |
| Kinurenine | Kinurenine | 62.28±0.68 | 0.00 | 9.95E-14 | 5.11 | y = 0.00529 x - 0.20693 | 0.99923 |
| Kynurenic Acid | Kynurenic_Acid | 25.26±2.25 | 0.00 | 3.62E-04 | 3.54 | y = 0.05136 x | 0.99673 |
| L-Citrulline | L_Citrulline | 2443.00±157.11 | 166.63±28.71 | 1.45E-04 | 9.32 | y = 3.26472e-4 x - 0.01283 | 0.99212 |
| L-Cystine | L_Cystine | 10092.00±736.21 | 62.91±45.99 | 1.74E-04 | 10.77 | y = 2.75431e-5 x + 7.82942e-4 | 0.99742 |
| L-Homocitrulline | L_Homocitrulline | 73.45±1.09 | 0.00 | 2.80E-10 | 9.08 | y = 0.00342 x - 0.16766 | 0.99477 |
| L-Ornithine | L_Ornithine | 1511.20±18.06 | 940.34±10.05 | 1.27E-05 | 10.45 | y = 5.65524e-4 x + 0.01828 | 0.99909 |
| L-Pipecolic Acid | L_Pipecolic_Acid | 45.84±0.56 | 33.79±0.19 | 4.22E-05 | 6.96 | y = 0.03978 x - 0.98505 | 0.99697 |
| L-Leucine | Leu | 3543.40±246.59 | 14.11±0.05 | 1.43E-04 | 4.98 | y = 0.00805 x - 0.02408 | 0.99957 |
| L-Lysine | Lys | 9118.30±442.94 | 171.36±15.87 | 4.29E-05 | 10.41 | y = 0.00414 x - 0.05058 | 0.99855 |
| L-Methionine | Met | 102.81±2.30 | 0.00 | 3.26E-07 | 6.04 | y = 0.00226 x + 0.06023 | 0.99913 |
| Methionine Sulfoxide | Methionine_Sulfoxide | 104.79±1.93 | 0.00 | 2.18E-08 | 8.90 | y = 0.00363 x - 0.16095 | 0.99611 |
| N,N-Dimethylglycine | N,N_Dimethylglycine | 49.17±0.66 | 41.48±0.22 | 3.89E-04 | 6.37 | y = 0.02225 x - 0.66632 | 0.99782 |
| N-Acetylneuraminic Acid | N_Acetylneuraminic_Acid | 5640.60±463.43 | 447.47±60.38 | 3.76E-04 | 9.53 | y = 1.19427e-4 x - 0.00521 | 0.9952 |
| N-Glycyl-L-Leucine | N_Glycyl_L_Leucine | 62.61±2.49 | 0.00 | 1.93E-05 | 7.36 | y = 8.76887e-4 x - 0.02201 | 0.99789 |
| N-Isovaleroylglycine | N_Isovaleroylglycine | 32.34±0.26 | 32.39±0.09 | 8.82E-01 | 1.45 | y = 0.00200 x - 0.04773 | 0.99659 |
| N-Propionylglycine | N_Propionylglycine | 36.31±0.54 | 37.89±1.14 | 3.89E-04 | 2.45 | y = 6.72678e-4 x - 0.01611 | 0.9967 |
| N6-Acetyl-L-Lysine | N6_Acetyl_L_Lysine | 23.78±0.98 | 0.00 | 2.79E-01 | 8.30 | y = 0.01460 x - 0.05978 | 0.99963 |
| N8-Acetylspermidine | N8_Acetylspermidine | 203.54±21.68 | 0.00 | 2.19E-05 | 9.67 | y = 8.56063e-4 x - 0.05005 | 0.99605 |
| O-Phospho-L-Serine | O_Phospho_L_Serine | 9501.20±397.98 | 0.00 | 2.36E-05 | 10.85 | y = 1.21012e-4 x - 0.07603 | 0.9923 |
| L-Phenylalanine | Phe | 984.80±56.51 | 54.83±0.16 | 8.61E-05 | 4.92 | y = 0.00958 x - 0.38377 | 0.99903 |
| L-Proline | Pro | 2555.00±138.05 | 56.27±1.14 | 6.19E-05 | 6.67 | y = 0.00325 x - 0.09946 | 0.99963 |
| S-(5-Adenosyl)-L-Homocysteine | S_(5_Adenosyl)_L_Homocysteine | 410.67±34.31 | 0.00 | 2.82E-04 | 9.31 | y = 0.00125 x - 0.03878 | 0.99635 |
| S-Sulfo-L-Cysteine | S_Sulfo_L_Cysteine | 118.28±31.39 | 0.00 | 1.96E-02 | 7.98 | y = 1.60998e-4 x + 0.00333 | 0.99757 |
| Sarcosine | Sarcosine | 51.50±2.33 | 0.00 | 3.13E-05 | 7.66 | y = 0.00149 x - 0.03962 | 0.99737 |
| L-Serine | Ser | 3011.00±281.06 | 0.00 | 4.33E-04 | 8.99 | y = 7.01440e-5 x - 0.00140 | 0.99891 |
| Succinic Acid | Succinic_Acid | 15859.00±752.23 | 2017.80±511.22 | 1.14E-04 | 1.50 | y = 3.38627e-6 x + 0.00523 | 0.99358 |
| L-Threonine | Thr | 3514.00±165.12 | 62.91±7.03 | 3.82E-05 | 8.39 | y = 8.07349e-4 x - 0.02041 | 0.99882 |
| Trans-4-Hydroxy-L-Proline | Trans_4_Hydroxy_L_Proline | 523.22±22.46 | 82.73±1.17 | 4.75E-05 | 8.11 | y = 0.00693 x - 0.27352 | 0.99871 |
| Trimethylamine N-Oxide | Trimethylamine_N_Oxide | 80.40±7.56 | 15.33±0.35 | 1.01E-03 | 3.29 | y = 0.16887 x | 0.99085 |
| L-Tryptophan | Trp | 7669.30±281.45 | 0.00 | 1.35E-05 | 5.11 | y = 2.71789e-5 x + 2.15955e-5 | 0.9976 |
| L-Tyrosine | Tyr | 1502.30±86.69 | 55.54±0.18 | 8.19E-05 | 7.08 | y = 0.00486 x - 0.16607 | 0.99937 |
| Urea | Urea | 114663.00±3388.20 | 3325.60±221.75 | 4.84E-06 | 1.57 | y = 6.17762e-4 x + 0.04366 | 0.99339 |
| L-Valine | Val | 2946.60±169.80 | 58.45±0.30 | 7.66E-05 | 6.65 | y = 0.00737 x - 0.19366 | 0.99913 |
| α-Aminoadipic acid | α_Aminoadipic_acid | 1774.80±78.64 | 0.00 | 2.92E-05 | 8.92 | y = 2.14468e-4 x - 0.01306 | 0.99815 |

**Note:** The amino acid concentration is expressed as Mean ± SE.

**Table S4. Different amino acids of concentrations in mice quadriceps after EVs injection**

| **Amino acids**  **(Compounds: full name)** | **Amino acids**  **(Index: abbreviation)** | **Concentrations (ng/g)** | | | ***P* values** | | | **RT** | **Standard curves** | **r** |
| --- | --- | --- | --- | --- | --- | --- | --- | --- | --- | --- |
|  |  | **Control** | **HME group** | **BME group** | **HME vs Control** | **BME vs Control** | **HME vs BME** |  |  |  |
| (S)_β_Aminoisobutyric Acid | (S)_β_Aminoisobutyric_Acid | 1021.90±73.07 | 1196.60±91.36 | 1148.90±135.12 | 0.47 | 0.67 | 0.94 | 7.39 | y = 1.58543e-4 x - 0.00596 | 0.99974 |
| 1_Methylhistidine | 1_Methylhistidine | 8682.70±861.06 | 9554.00±409.88 | 9547.70±733.39 | 0.66 | 0.66 | 1.00 | 9.35 | y = 0.02095 x - 1.66538 | 0.99258 |
| 2_Aminobutyric acid | 2_Aminobutyric_acid | 674.97±33.73 | 798.84±83.87 | 685.94±37.62 | 0.30 | 0.99 | 0.36 | 7.57 | y = 0.00365 x - 0.07727 | 0.99827 |
| 2_Aminoethanesulfonic Acid | 2_Aminoethanesulfonic_Acid | 1014440.00±44886.00 | 1167909.00±94834.00 | 1032053.00±64832.00 | 0.31 | 0.98 | 0.39 | 7.17 | y = 0.00541 x - 0.13652 | 0.99783 |
| 3_Aminoisobutanoic Acid | 3_Aminoisobutanoic_Acid | 6097.50±650.55 | 5349.60±581.30 | 5289.60±608.99 | 0.67 | 0.63 | 1.00 | 7.39 | y = 1.63464e-4 x + 0.00380 | 0.99493 |
| 3_Hydroxyhippuric Acid | 3_Hydroxyhippuric_Acid | 86.20±69.43 | 25.64±12.63 | 20.83±6.74 | 0.58 | 0.60 | 1.00 | 2.68 | y = 0.00325 x | 0.99717 |
| 3_N_Methyl_L_Histidine | 3_N_Methyl_L_Histidine | 14594.00±973.23 | 14678.00±559.70 | 15751.00±1266.90 | 1.00 | 0.69 | 0.72 | 10.05 | y = 0.00318 x - 0.14133 | 0.99895 |
| 4_Acetamidobutyric Acid | 4_Acetamidobutyric_Acid | 309.90±45.97 | 419.05±40.75 | 388.58±30.09 | 0.16 | 0.36 | 0.85 | 1.33 | y = 0.00410 x - 0.01513 | 0.99795 |
| 5_Hydroxy_Tryptamine | 5_Hydroxy_Tryptamine | 25829.00±10378.00 | 10162.00±6349.50 | 16065.00±6981.10 | 0.50 | 0.87 | 0.81 | 2.97 | y = 4.96660e-5 x + 0.05798 | 0.99890 |
| 5_Hydroxylysine | 5_Hydroxylysine | 771.85±71.89 | 951.99±97.94 | 740.50±70.12 | 0.29 | 0.96 | 0.19 | 10.56 | y = 5.31170e-4 x - 0.01565 | 0.99811 |
| (5_L_Glutamyl)_L_Amino Acid | 5_L_Glutamyl_L_Amino_Acid | 2172.70±674.14 | 4628.70±839.51 | 2717.70±1359.30 | 0.22 | 0.74 | 0.63 | 9.72 | y = 2.60626e-4 x + 0.00215 | 0.99748 |
| L_Alanine | Ala | 726174.00±32869.00 | 812063.00±51036.00 | 735118.00±54702.00 | 0.42 | 0.99 | 0.50 | 8.14 | y = 7.00977e-4 x + 0.03767 | 0.99004 |
| Anserine | Anserine | 3548522.00±147162.00 | 4202127.00±326785.00 | 3835704.00±401725.00 | 0.32 | 0.79 | 0.69 | 10.29 | y = 7.59813e-4 x - 0.06765 | 0.99766 |
| L_Arginine | Arg | 95217.00±8097.90 | 83730.00±6603.10 | 81068.00±6482.70 | 0.50 | 0.36 | 0.96 | 10.13 | y = 0.00628 x - 0.34310 | 0.99678 |
| argininosuccinic acid | argininosuccinic_acid | 33465.00±4488.60 | 31923.00±1705.50 | 40244.00±5463.70 | 0.96 | 0.50 | 0.37 | 10.76 | y = 2.33842e-5 x - 0.01308 | 0.99173 |
| L_Asparagine Anhydrous | Asn | 16239.00±979.17 | 20907.00±1668.40 | 22902.00±1675.20 | 0.10 | 0.02 | 0.62 | 9.04 | y = 6.93353e-4 x - 0.02473 | 0.99877 |
| L_Aspartate | Asp | 325020.00±36671.00 | 290101.00±19962.00 | 303526.00±27473.00 | 0.68 | 0.86 | 0.94 | 9.83 | y = 3.41914e-4 x + 0.01017 | 0.99895 |
| L_α_Aspartyl_L_phenylalanine | Asp_Phe | 830.33±118.93 | 1031.30±104.44 | 645.77±119.63 | 0.45 | 0.51 | 0.07 | 8.67 | y = 0.00125 x + 0.02616 | 0.99298 |
| Beta_Alanine | Beta_Alanine | 11849.00±1314.20 | 12183.00±1201.60 | 11501.00±1263.00 | 0.98 | 0.98 | 0.92 | 8.18 | y = 4.79772e-5 x - 2.37806e-4 | 0.99855 |
| Creatine | Creatine | 749756.00±42806.00 | 902228.00±87333.00 | 794413.00±67949.00 | 0.29 | 0.89 | 0.52 | 8.14 | y = 0.00904 x - 0.27868 | 0.99822 |
| Creatine Phosphate | Creatine_Phosphate | 906553.00±243554.00 | 670613.00±222738.00 | 904413.00±374552.00 | 0.83 | 1.00 | 0.84 | 10.73 | y = 7.66350e-7 x + 0.00564 | 0.99163 |
| L_Cysteine | Cys | 2100.00±941.10 | 0.00 | 2562.90±1148.80 | - | 0.01 | - | 7.49 | y = 2.06593e-4 x - 0.03261 | 0.99093 |
| D_Alanyl_D_Alanine | D_Alanyl_D_Alanine | 330.11±62.44 | 347.68±75.74 | 233.68±62.61 | 0.98 | 0.86 | 0.76 | 8.74 | y = 9.44581e-4 x - 0.01370 | 0.99981 |
| D_Homocysteine | D_Homocysteine | 269.61±120.82 | 0.00 | 281.19±128.57 | - | 0.73 | - | 6.83 | y = 5.98736e-4 x - 0.01455 | 0.99962 |
| Ethanolamine | Ethanolamine | 3186.20±210.50 | 4041.40±631.49 | 4670.30±347.63 | 0.37 | 0.07 | 0.57 | 5.37 | y = 8.02998e-4 x + 0.03754 | 0.99704 |
| L_Glutamine | Gln | 1043297.00±81156.00 | 1254402.00±58313.00 | 1086075.00±74414.00 | 0.13 | 0.91 | 0.25 | 8.89 | y = 5.15643e-4 x - 0.02262 | 0.99431 |
| L_Glutamic acid | Glu | 341748.00±60187.00 | 367322.00±20647.00 | 369459.00±27687.00 | 0.89 | 0.88 | 1.00 | 9.32 | y = 3.85226e-4 x + 0.01614 | 0.99463 |
| Glutathione Oxidized | Glutathione_Oxidized | 3846338.00±454143.00 | 4594138.00±529038.00 | 5366752.00±626550.00 | 0.60 | 0.15 | 0.58 | 11.03 | y = 3.10840e-6 x + 0.02449 | 0.99202 |
| Glycine | Gly | 185585.00±10428.00 | 181453.00±12134.00 | 150198.00±9558.50 | 0.96 | 0.08 | 0.13 | 8.57 | y = 4.21547e-5 x + 0.00231 | 0.99833 |
| Glycyl_L_Proline | Glycyl_L_Proline | 378.19±27.39 | 482.50±48.08 | 449.35±35.13 | 0.16 | 0.40 | 0.81 | 8.99 | y = 0.01487 x - 0.36031 | 0.99854 |
| glycylphenylalanine | glycylphenylalanine | 477.65±10.74 | 558.92±56.23 | 515.49±36.85 | 0.34 | 0.78 | 0.72 | 7.42 | y = 0.00378 x - 0.10202 | 0.99804 |
| Guanidinoethyl Sulfonate | Guanidinoethyl_Sulfonate | 2499.30±92.93 | 2856.00±202.56 | 2574.10±310.71 | 0.50 | 0.97 | 0.65 | 7.02 | y = 0.00262 x - 0.03212 | 0.99775 |
| L_Histidine | His | 93039.00±4512.70 | 112098.00±10498.00 | 112880.00±8417.80 | 0.26 | 0.23 | 1.00 | 9.94 | y = 0.00147 x - 0.09597 | 0.99791 |
| Homo_L_arginine | Homo_Arg | 5008.00±353.17 | 6275.20±599.44 | 4795.30±554.03 | 0.22 | 0.95 | 0.14 | 9.98 | y = 0.00831 x - 0.38932 | 0.99875 |
| Homoserine | Homoserine | 1416.90±215.42 | 1843.40±82.20 | 1791.00±149.11 | 0.17 | 0.25 | 0.97 | 8.54 | y = 7.02506e-4 x - 0.01930 | 0.99908 |
| L_Isoleucine | Ile | 21315.00±1518.10 | 22362.00±722.38 | 25277.00±1759.60 | 0.86 | 0.15 | 0.33 | 5.41 | y = 0.00836 x - 0.18023 | 0.99882 |
| Kinurenine | Kinurenine | 446.68±19.49 | 580.31±63.84 | 564.86±43.00 | 0.13 | 0.20 | 0.97 | 5.17 | y = 0.00529 x - 0.20693 | 0.99923 |
| Kynurenic Acid | Kynurenic_Acid | 5.15±2.84 | 1.85±1.27 | 2.25±1.04 | 0.92 | 0.87 | 1.00 | 3.59 | y = 0.05136 x | 0.99673 |
| L_Carnosine | L_Carnosine | 2685634.00±119303.00 | 3136706.00±296752.00 | 2737093.00±259436.00 | 0.40 | 0.99 | 0.48 | 10.34 | y = 9.66722e-4 x - 0.04185 | 0.99856 |
| L_Citrulline | L_Citrulline | 102047.00±12401.00 | 99627.00±8826.90 | 69247.00±4624.80 | 0.98 | 0.06 | 0.08 | 9.24 | y = 3.26472e-4 x - 0.01283 | 0.99212 |
| L_Cystathionine | L_Cystathionine | 1041.80±113.23 | 1262.90±100.03 | 1184.60±195.97 | 0.53 | 0.76 | 0.92 | 10.65 | y = 6.39224e-4 x - 0.01000 | 0.99723 |
| L_Cystine | L_Cystine | 33.06±21.17 | 0.00 | 85.44±56.38 | - | 0.00 | - | 10.70 | y = 2.75431e-5 x + 7.82942e-4 | 0.99742 |
| L_Homocitrulline | L_Homocitrulline | 731.55±22.89 | 877.17±69.57 | 793.06±69.62 | 0.21 | 0.74 | 0.58 | 9.01 | y = 0.00342 x - 0.16766 | 0.99477 |
| L_Ornithine | L_Ornithine | 16813.00±3012.50 | 66042.00±6185.50 | 37416.00±5857.90 | 0.00 | 0.03 | 0.00 | 10.33 | y = 5.65524e-4 x + 0.01828 | 0.99909 |
| L_Pipecolic Acid | L_Pipecolic_Acid | 1028.40±130.80 | 850.04±75.18 | 861.65±84.76 | 0.44 | 0.48 | 1.00 | 7.03 | y = 0.03978 x - 0.98505 | 0.99697 |
| L_Theanine | L_Theanine | 217.08±98.00 | 422.99±177.81 | 311.33±148.07 | 0.61 | 0.67 | 1.00 | 7.42 | y = 0.00743 x - 0.29337 | 0.99833 |
| L_tyrosine methyl ester | L_tyrosine_methyl_ester | 17.84±1.62 | 29.56±4.21 | 33.26±4.92 | 0.11 | 0.03 | 0.78 | 1.58 | y = 0.02233 x | 0.99584 |
| L_Leucine | Leu | 33460.00±2236.70 | 37090.00±1860.60 | 41949.00±3205.10 | 0.57 | 0.07 | 0.38 | 5.10 | y = 0.00805 x - 0.02408 | 0.99957 |
| L_Lysine | Lys | 90625.00±10191.00 | 110103.00±7271.30 | 84871.00±9275.70 | 0.31 | 0.89 | 0.15 | 10.28 | y = 0.00414 x - 0.05058 | 0.99855 |
| L_Methionine | Met | 12843.00±828.41 | 16412.00±1557.50 | 16646.00±1422.30 | 0.16 | 0.13 | 0.99 | 6.20 | y = 0.00209 x + 0.10773 | 0.99721 |
| Methionine Sulfoxide | Methionine_Sulfoxide | 767.46±37.98 | 986.97±95.95 | 886.98±96.26 | 0.17 | 0.57 | 0.67 | 8.85 | y = 0.00363 x - 0.16095 | 0.99611 |
| N_Acetyl_L_Tyrosine | N_Acetyl_L_Tyrosine | 286.49±13.00 | 348.56±37.33 | 336.86±29.69 | 0.30 | 0.44 | 0.95 | 2.28 | y = 0.00303 x - 0.06649 | 0.99532 |
| N_Acetylaspartate | N_Acetylaspartate | 4549.60±580.17 | 3948.20±245.13 | 5367.30±685.24 | 0.71 | 0.54 | 0.18 | 5.62 | y = 1.80058e-4 x + 8.31967e-4 | 0.99689 |
| N_Acetylneuraminic Acid | N_Acetylneuraminic_Acid | 12043.00±2049.70 | 22238.00±3252.60 | 12780.00±3645.10 | 0.08 | 0.98 | 0.11 | 9.50 | y = 1.19427e-4 x - 0.00521 | 0.99520 |
| N'_Formylkynurenine | N_Formylkynurenine | 596.21±77.55 | 583.06±74.28 | 936.75±118.56 | 0.99 | 0.05 | 0.04 | 7.15 | y = 3.56515e-4 x + 0.00213 | 0.99784 |
| N_Glycyl_L_Leucine | N_Glycyl_L_Leucine | 2966.90±354.53 | 2709.30±348.67 | 2531.60±398.10 | 0.87 | 0.69 | 0.94 | 7.45 | y = 8.76887e-4 x - 0.02201 | 0.99789 |
| N_Isovaleroylglycine | N_Isovaleroylglycine | 1401.80±739.37 | 977.00±408.30 | 667.17±121.52 | 0.82 | 0.56 | 0.90 | 1.62 | y = 0.00200 x - 0.04773 | 0.99659 |
| N,N_Dimethylglycine | N_N_Dimethylglycine | 869.27±55.07 | 1030.20±79.50 | 1029.20±49.95 | 0.20 | 0.20 | 1.00 | 6.54 | y = 0.02225 x - 0.66632 | 0.99782 |
| N_Propionylglycine | N_Propionylglycine | 399.03±63.38 | 434.34±51.00 | 396.49±43.00 | 0.89 | 1.00 | 0.87 | 2.84 | y = 6.72678e-4 x - 0.01611 | 0.99670 |
| N6_Acetyl_L_Lysine | N6_Acetyl_L_Lysine | 299.23±18.26 | 333.77±11.99 | 336.88±21.21 | 0.37 | 0.31 | 0.99 | 8.28 | y = 0.01460 x - 0.05978 | 0.99963 |
| N8_Acetylspermidine | N8_Acetylspermidine | 1563.60±107.88 | 1837.00±123.81 | 1569.70±147.55 | 0.31 | 1.00 | 0.33 | 9.61 | y = 8.56063e-4 x - 0.05005 | 0.99605 |
| Nα_Acetyl_L_Arginine | Nα_Acetyl_L_Arginine | 960.62±49.93 | 994.83±57.09 | 880.71±71.94 | 0.92 | 0.63 | 0.40 | 8.40 | y = 0.00530 x - 0.14909 | 0.99879 |
| Nα_Acetyl_L_glutamine | Nα_Acetyl_L_glutamine | 1126.60±413.83 | 1138.60±133.39 | 1176.10±288.61 | 0.84 | 0.99 | 0.75 | 7.27 | y = 2.47125e-4 x - 0.00433 | 0.99898 |
| L_Phenylalanine | Phe | 19186.00±1563.70 | 21897.00±1285.60 | 23670.00±1757.20 | 0.45 | 0.13 | 0.70 | 5.00 | y = 0.00958 x - 0.38377 | 0.99903 |
| L_Proline | Pro | 35760.00±1856.90 | 44366.00±3628.30 | 45379.00±1676.30 | 0.07 | 0.04 | 0.96 | 6.82 | y = 0.00325 x - 0.09946 | 0.99963 |
| S_(5_Adenosyl)_L_Homocysteine | S_5_Adenosyl_L_Homocysteine | 5368.50±775.11 | 2706.30±304.93 | 3457.10±413.52 | 0.01 | 0.06 | 0.59 | 9.21 | y = 0.00125 x - 0.03878 | 0.99635 |
| Sarcosine | Sarcosine | 771.97±35.14 | 768.44±53.86 | 616.96±46.10 | 1.00 | 0.07 | 0.08 | 7.71 | y = 0.00149 x - 0.03962 | 0.99737 |
| L_Serine | Ser | 41682.00±3917.20 | 46322.00±3578.70 | 48981.00±4017.70 | 0.68 | 0.39 | 0.88 | 8.95 | y = 7.01440e-5 x - 0.00140 | 0.99891 |
| Succinic Acid | Succinic_Acid | 568288.00±347539.00 | 1509805.00±912983.00 | 1655493.00±872729.00 | 0.66 | 0.58 | 0.99 | 1.72 | y = 3.38627e-6 x + 0.00523 | 0.99358 |
| L_Threonine | Thr | 47846.00±3326.70 | 52772.00±2899.50 | 57174.00±3261.30 | 0.53 | 0.13 | 0.60 | 8.39 | y = 8.07349e-4 x - 0.02041 | 0.99882 |
| Trans_4_Hydroxy_L_Proline | Trans_4_Hydroxy_L_Proline | 37653.00±1525.80 | 41873.00±2705.90 | 36914.00±2814.10 | 0.45 | 0.97 | 0.34 | 8.13 | y = 0.00693 x - 0.27352 | 0.99871 |
| Trimethylamine N_Oxide | Trimethylamine_N_Oxide | 911.77±111.70 | 890.61±115.94 | 585.24±93.48 | 0.99 | 0.11 | 0.14 | 3.34 | y = 0.16887 x | 0.99085 |
| L_Tryptophan | Trp | 53340.00±4882.70 | 46281.00±5793.00 | 44194.00±10126.00 | 0.78 | 0.66 | 0.98 | 5.15 | y = 2.71789e-5 x + 2.15955e-5 | 0.99760 |
| L_Tryptophyl_L_glutamic acid | TRP_GLU | 8247.00±2700.60 | 3465.60±1172.60 | 7216.40±2854.00 | 0.35 | 0.96 | 0.27 | 8.27 | y = 1.74623e-5 x + 7.88082e-5 | 0.99670 |
| L_Tyrosine | Tyr | 45431.00±3103.10 | 52281.00±5514.50 | 52005.00±2671.20 | 0.46 | 0.49 | 1.00 | 7.14 | y = 0.00486 x - 0.16607 | 0.99937 |
| Urea | Urea | 444564.00±24436.00 | 351747.00±34898.00 | 321186.00±23604.00 | 0.08 | 0.02 | 0.73 | 1.59 | y = 6.17762e-4 x + 0.04366 | 0.99339 |
| L_Valine | Val | 32001.00±2038.40 | 33669.00±1682.80 | 37254.00±2497.00 | 0.84 | 0.21 | 0.47 | 6.80 | y = 0.00737 x - 0.19366 | 0.99913 |
| α_Aminoadipic acid | α_Aminoadipic_acid | 11421.00±1584.70 | 13380.00±741.99 | 13637.00±1396.90 | 0.55 | 0.46 | 0.99 | 8.93 | y = 2.14468e-4 x - 0.01306 | 0.99815 |
| γ_Aminobutyric Acid | γ_Aminobutyric_Acid | 19972.00±2920.90 | 10623.00±1577.60 | 11410.00±4062.80 | 0.04 | 0.73 | 0.23 | 7.29 | y = 4.11383e-5 x + 0.00379 | 0.99772 |
| γ_Glutamate_Cysteine | γ_Glutamate_Cysteine | 19215.00±2604.80 | 20939.00±1527.80 | 14913.00±2717.90 | 0.86 | 0.42 | 0.20 | 9.70 | y = 5.05280e-5 x - 0.00207 | 0.99728 |

**Note:** The amino acid concentration is expressed as Mean ± SE.
