# Supplementary material for: Human milk extracellular vesicles enhance muscle growth and physical performance of immature mice associating with Akt/mTOR/p70s6k signaling pathway
Source: J Nanobiotechnology. 2023 Aug 29;21:304. doi: 10.1186/s12951-023-02043-6 (PMC10463453; doi:10.1186/s12951-023-02043-6)
Supplement: Supplementary file 2 — Supplementary Material 2 [file 12951_2023_2043_MOESM2_ESM.docx]

**Supplemental Materials and methods S1**

**Targeted metabolomics analysis**

**1: PCA**

Unsupervised PCA (principal component analysis) was performed by statistics function prcomp within R (www.r-project.org). The data was unit variance scaled before unsupervised PCA.

**2: Hierarchical Cluster Analysis and Pearson Correlation Coefficients**

The HCA (hierarchical cluster analysis) results of samples and metabolites were presented as heatmaps with dendrograms, while Pearson correlation coefficients (PCC) between samples were calculated by the cor function in R and presented as only heatmaps. Both HCA and PCC were carried out by R package heatmaps. For HCA, normalized signal intensities of metabolites (unit variance scaling) are visualized as a color spectrum.

**3: Differential metabolites selected**

Significantly regulated metabolites between groups were determined by absolute Log2FC (fold change).

**4: KEGG annotation and enrichment analysis**

Identified metabolites were annotated using KEGG compound database (http://www.kegg.jp/kegg/compound/), annotated metabolites were then mapped to KEGG Pathway database (http://www.kegg.jp/kegg/pathway.html). Pathways with significantly regulated metabolites mapped to were then fed into MSEA (metabolite sets enrichment analysis), their significance was determined by hypergeometric test’s P-Values.
