# Supplementary material for: Human milk extracellular vesicles enhance muscle growth and physical performance of immature mice associating with Akt/mTOR/p70s6k signaling pathway
Source: J Nanobiotechnology. 2023 Aug 29;21:304. doi: 10.1186/s12951-023-02043-6 (PMC10463453; doi:10.1186/s12951-023-02043-6)
Supplement: Supplementary file 3 — Supplementary Material 3 [file 12951_2023_2043_MOESM3_ESM.docx]

**Supplemental Figures**


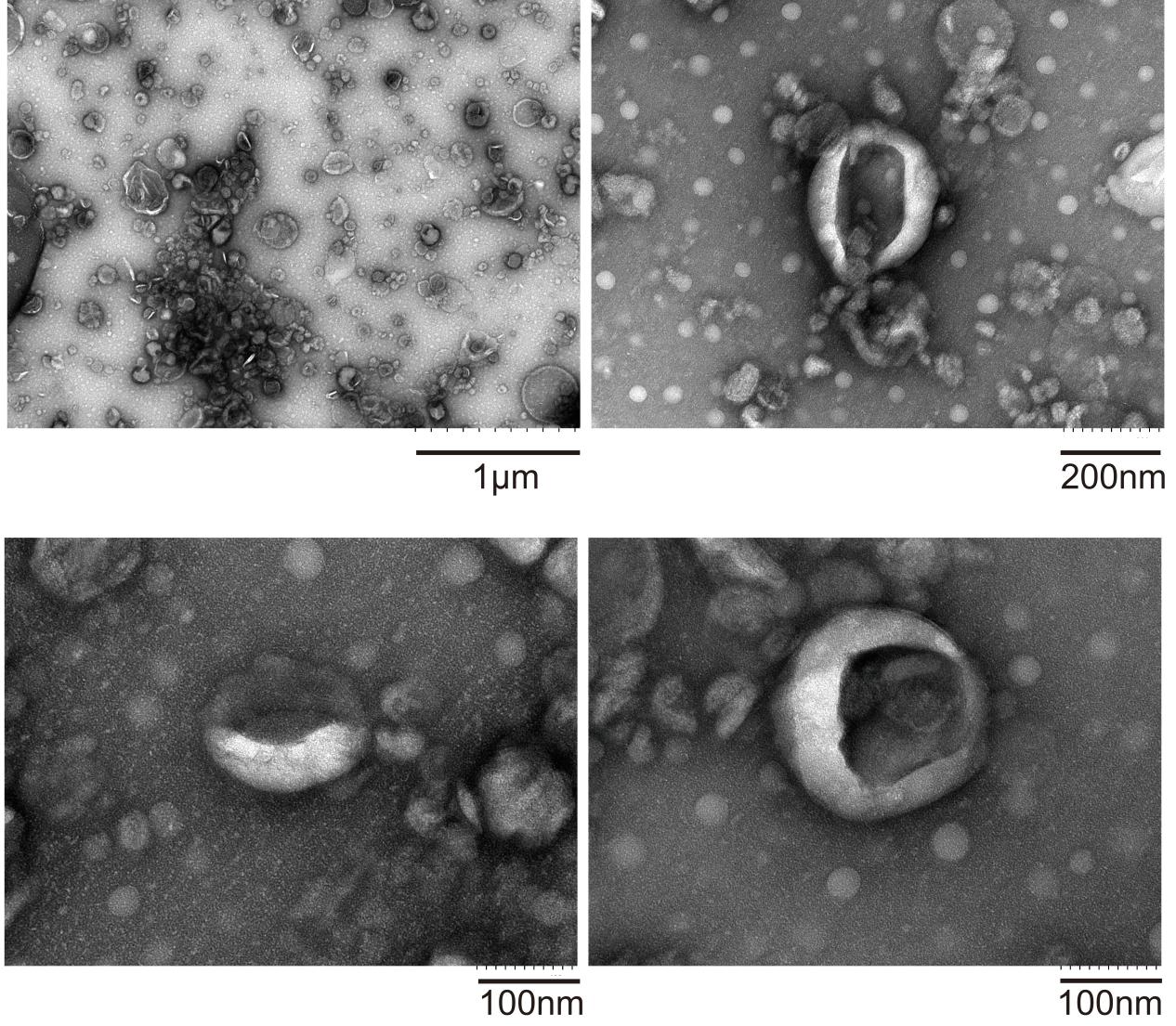


**Fig. S1.** Representative figures for morphology of HME by transmission electron microscopy (TEM).


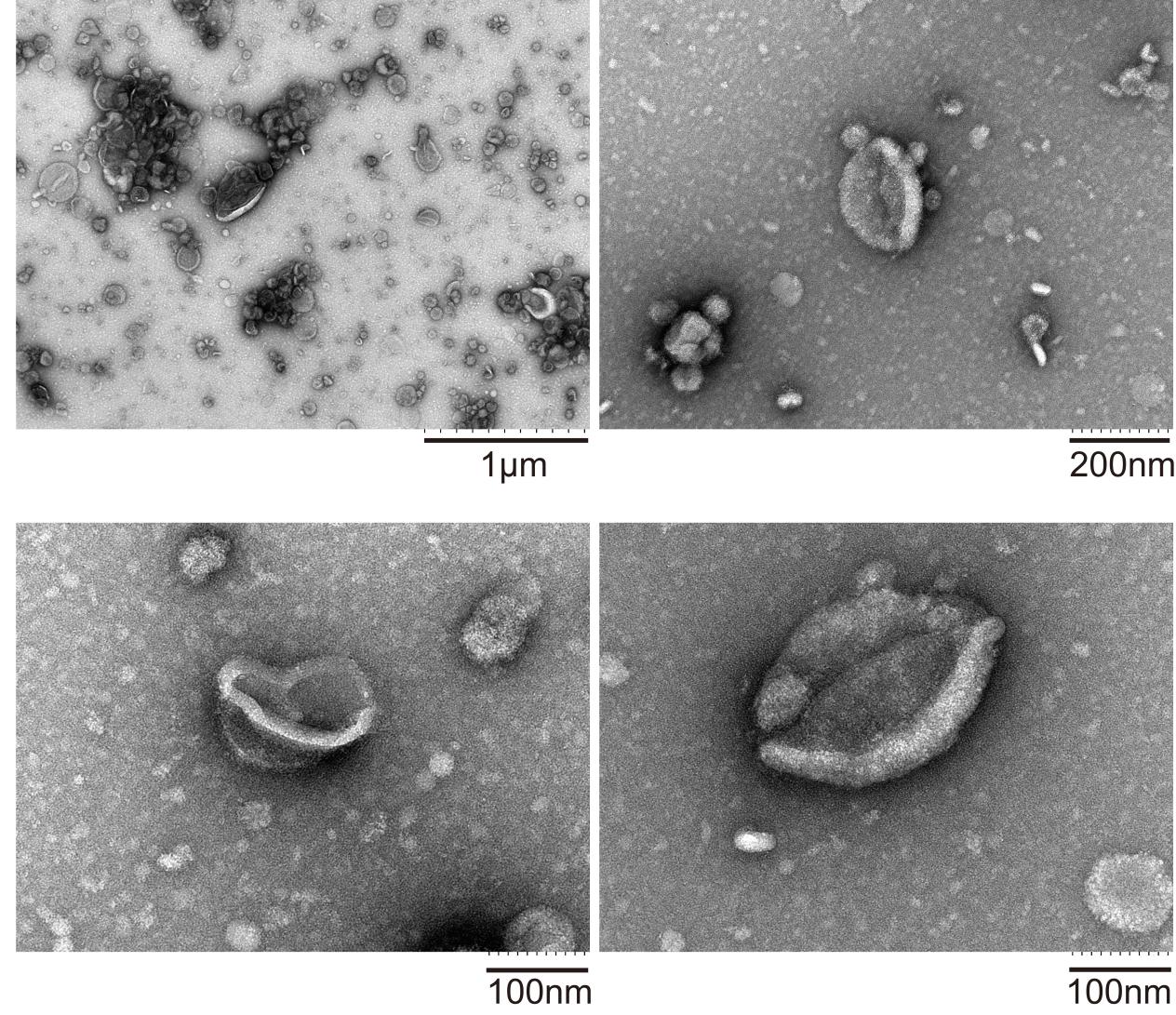


**Fig. S2.** Representative figures for morphology of BME by transmission electron microscopy (TEM).


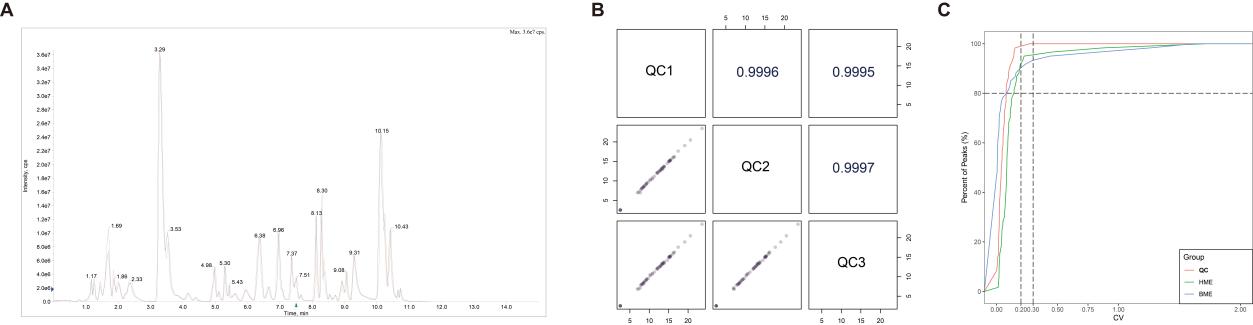


**Fig. S3.** Sample quality control and data analysis. (A): Total ion chromatogram (TIC) of amino acid profiles of HME and BME. (B): Quality control (QC) sample correlation. (C): Distribution of coefficient of variation (CV) values of all samples including QC samples.


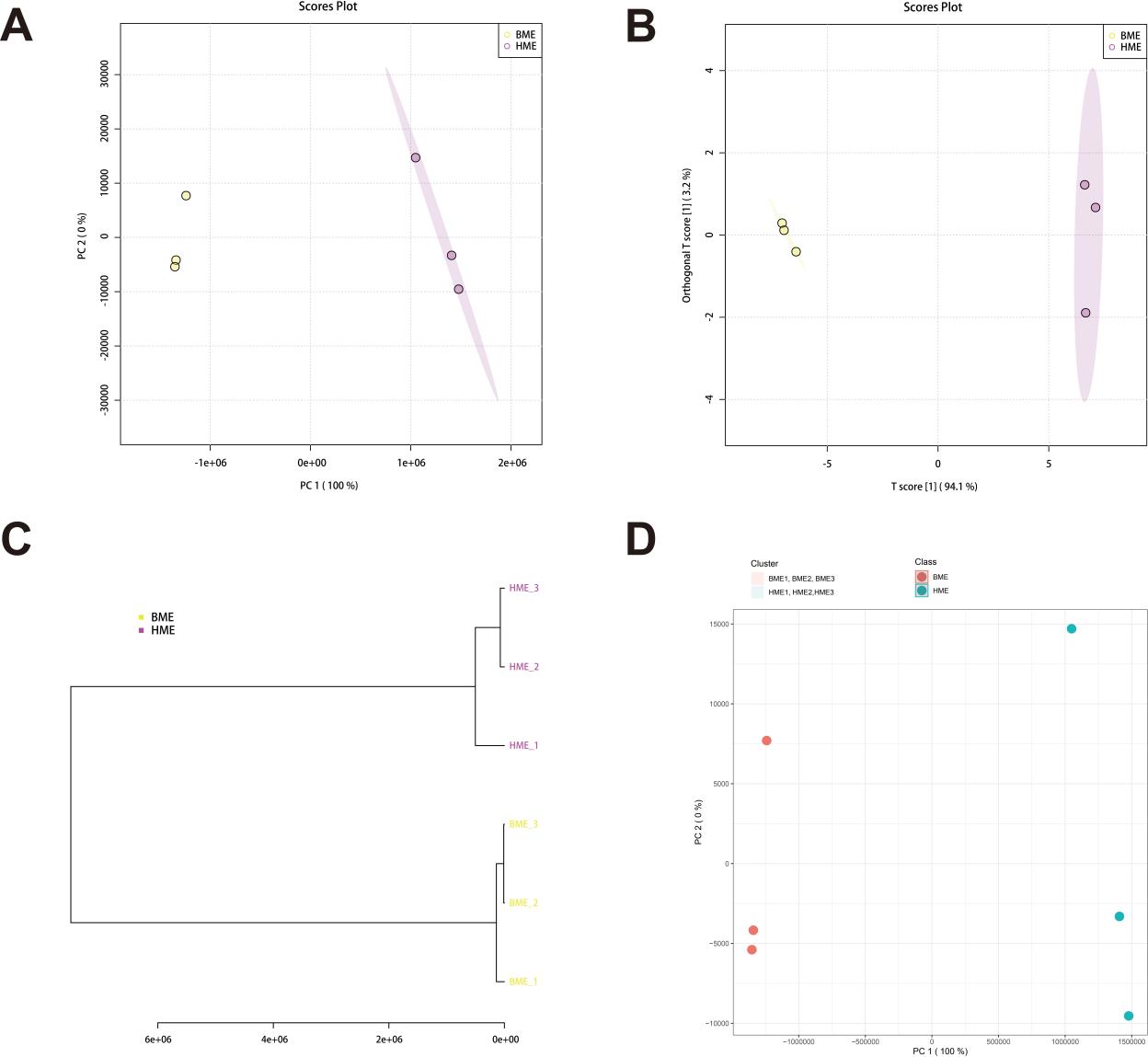


**Fig. S4.** Amino acid and metabolites profiles of HME and BME. (A): Principal component analysis (PCA) of all samples. (B): Orthogonal partial least squares discriminant analysis (OPLS-DA) of all samples. (C): Hierarchical clustering analysis of all samples. (D): Partitional clustering analysis of all samples.


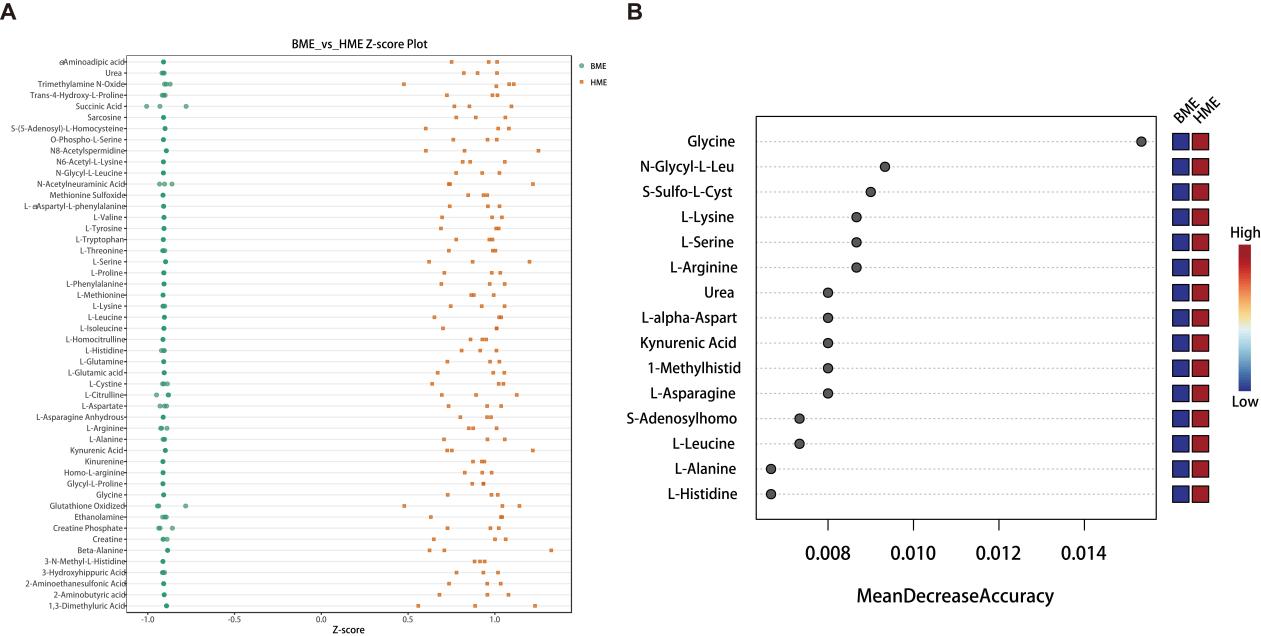


**Fig. S5.** Differential metabolites screening. (A): Z-score plot of different metabolites. (B): Random forest model prediction of the importance of metabolites.


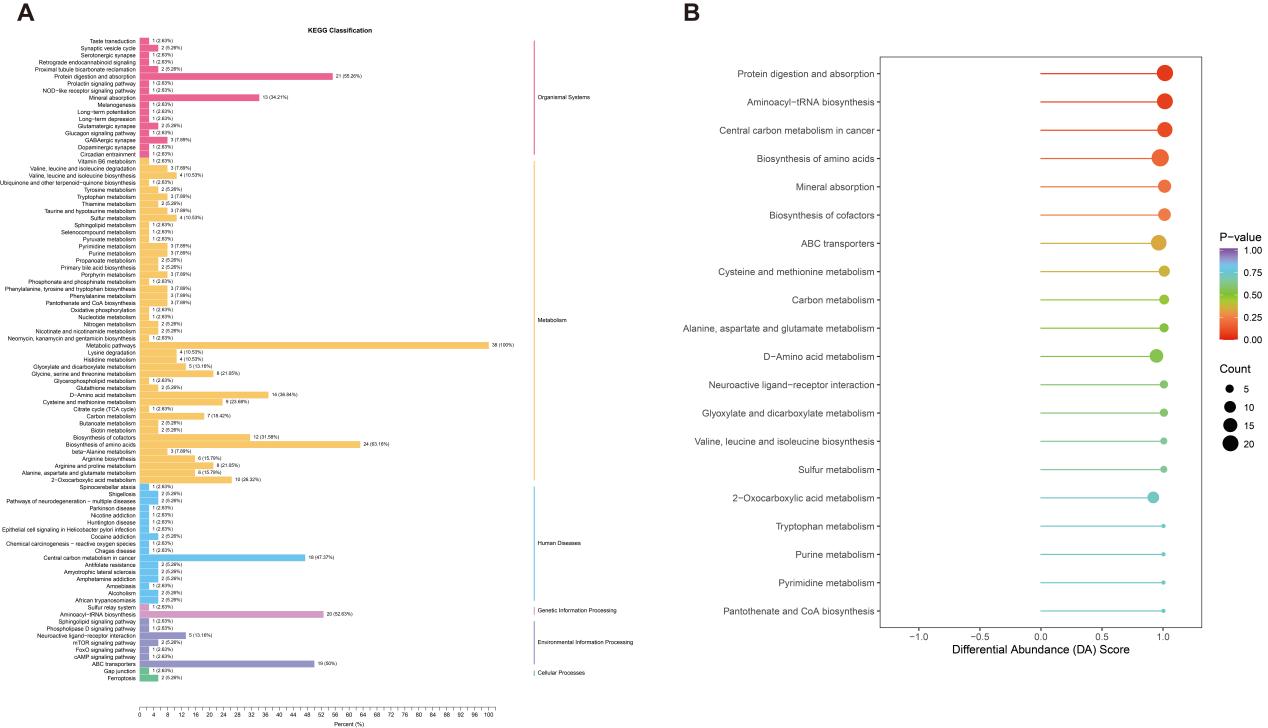


**Fig. S6.** KEGG annotation and enrichment analysis. (A): KEGG classification of different metabolites. (B): Differential abundance score plot of different metabolic pathways.


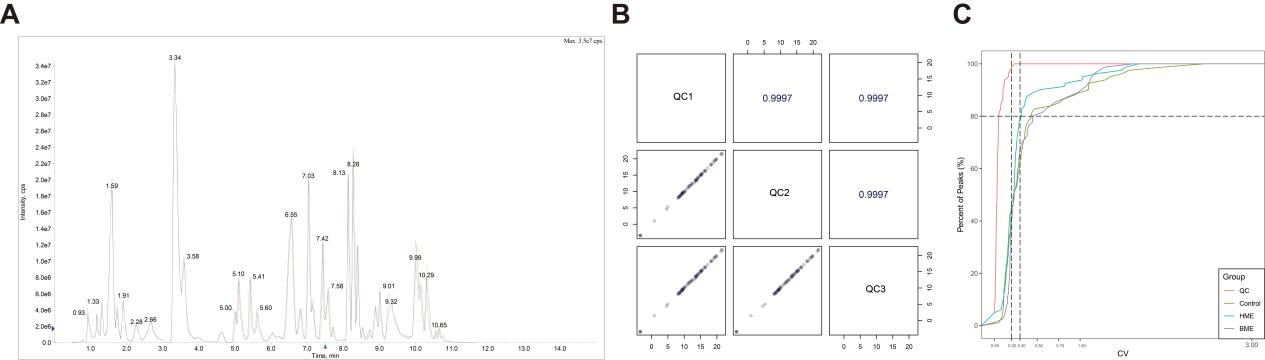


**Fig. S7.** Sample quality control and data analysis. (A): Total ion chromatogram (TIC) of amino acid profiling in quadriceps. (B): QC sample correlation. (C): Distribution of CV values including all samples and QC samples.


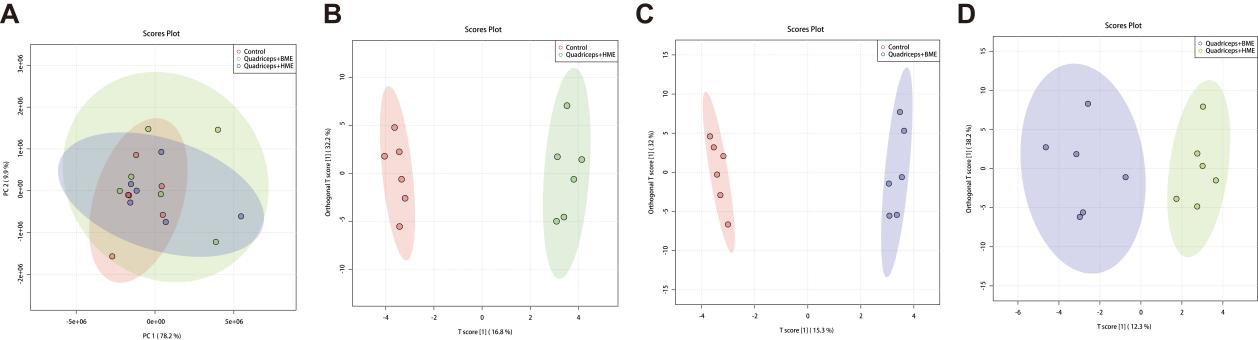


**Fig. S8.** Profiling of amino acid and metabolites in quadriceps from control, BME, and HME groups. (A): PCA analysis of all samples. (B-D): Orthogonal partial least squares discriminant analysis (OPLS-DA) of all samples.


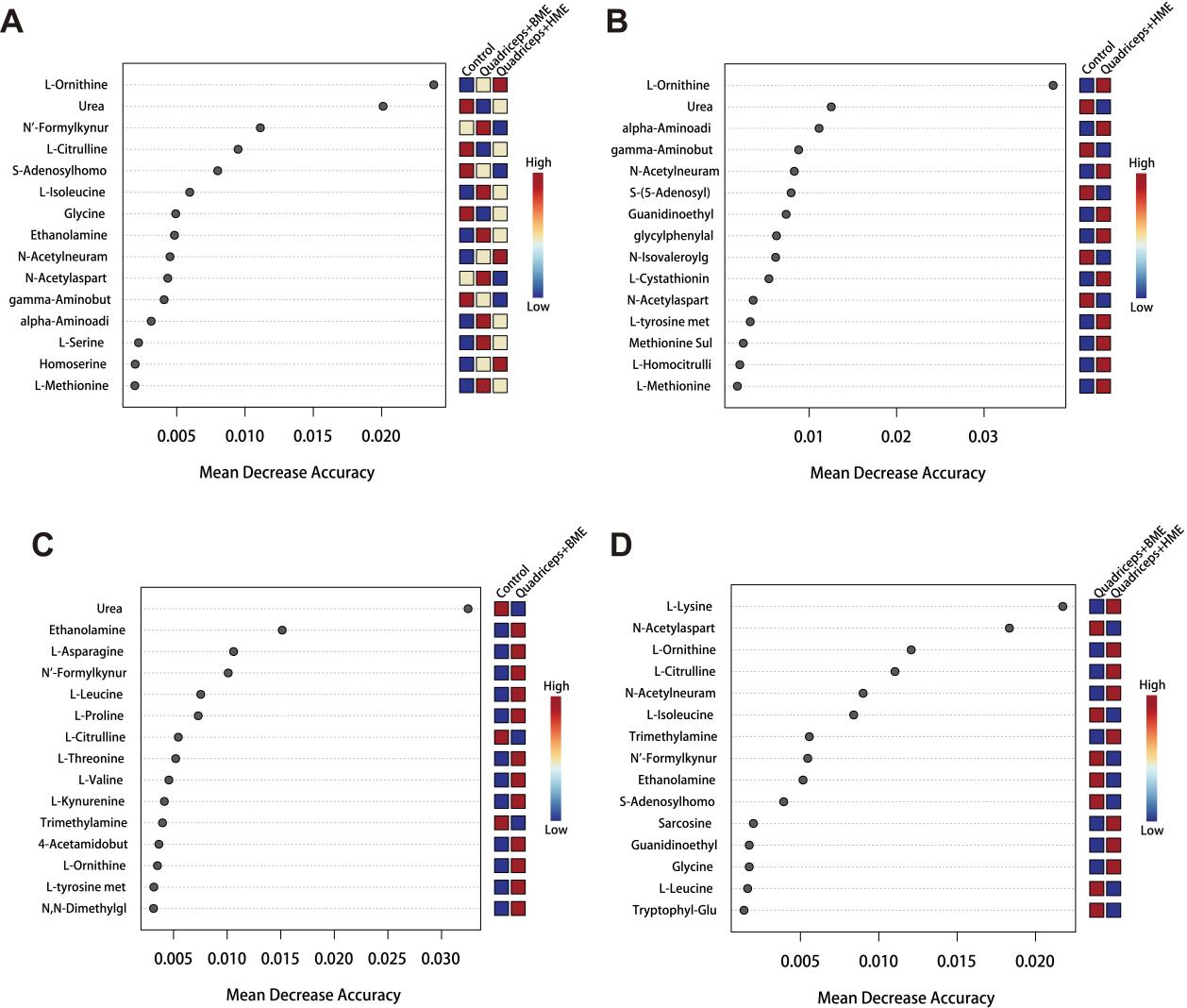


**Fig. S9.** Random forest model predicting the importance of metabolites (A-D).


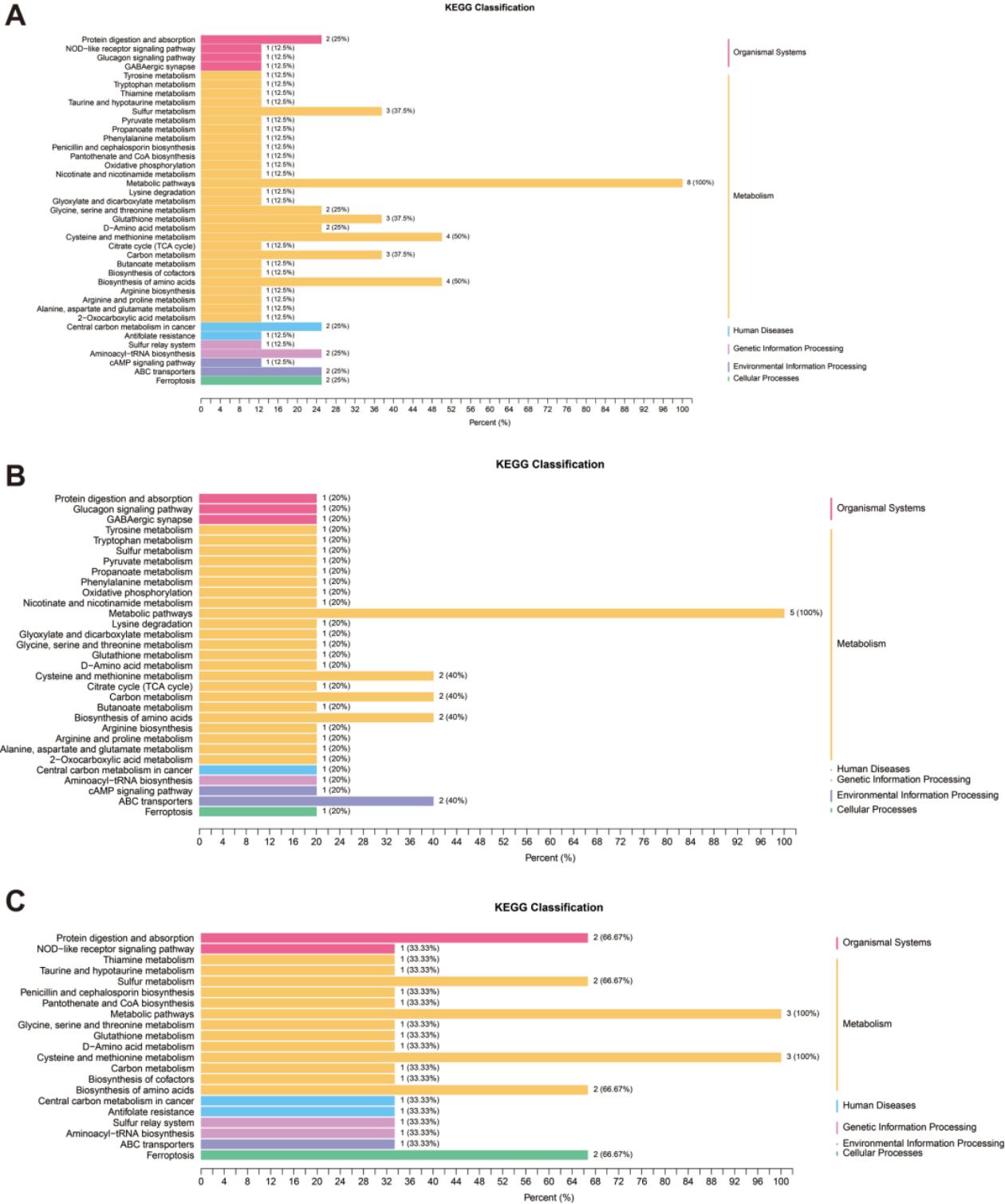


**Fig. S10.** KEGG annotation. (A): KEGG classification of differentially abundant metabolites between the HME and control groups. (B): KEGG classification of differentially abundant metabolites between the BME and control groups. (C): KEGG classification of differentially abundant metabolites between the HME and BME groups.
